# Multi‐Omics Analyses Identify Candidate Genes Associated With Stage‐Specific Quality Formation in Mulching‐Treated Moso Bamboo Shoots

**DOI:** 10.1002/advs.78312

**Published:** 2026-10-30

**Authors:** Bo Wang, Yanbin Wang, Chunyue Wang, Zishan Ahmad, Peixin Teng, Huijing Ni, Qiang Wei, Muthusamy Ramakrishnan

**Affiliations:** ^1^ Zhejiang Academy of Forestry Hangzhou Zhejiang China; ^2^ State Key Laboratory of Tree Genetics and Breeding Co‐Innovation Center for Sustainable Forestry in Southern China, Bamboo Research Institute, School of Life Sciences, Nanjing Forestry University Nanjing Jiangsu China; ^3^ Northwest Zhejiang Bamboo Forest Ecosystem Positioning Observation and Research Station National Forestry and Grassland Administration Hangzhou Zhejiang China

**Keywords:** bamboo shoots, moso bamboo, mulching stress, *Phyllostachys edulis*, rapid growth, spring shoots, winter shoots

## Abstract

Bamboo shoots are nutrient‐rich edible resources, and organic mulching promotes earlier shoot emergence and increases yield. However, the molecular mechanisms regulating mulching‐treated shoot (MtS) development remain unclear. Field experiments across three Moso bamboo forests, combined with biochemical profiling, metabolomics, and transcriptomics, examine MtS development. Mulching induces stage‐specific shoot quality formation (MtS1‐MtS4) and increases shoot size, yield, biochemical traits, and market value compared with spring shoots (SS) and winter shoots (WS). MtS increases soluble sugars, phenolics, tannins, and alkaloid‐related metabolites and reduces water content and antioxidant capacity. Metabolite profiling identifies 2029 metabolites and reveals stage‐dependent shifts in amino acid, carbohydrate, lipid, flavonoid, tricin‐related, and cell‐wall modification pathways. Transcriptome analysis identifies 13 691–18 038 differentially expressed genes across SS vs MtS comparisons. KEGG enrichment highlights sugar metabolism, lignin and cellulose synthesis, and hormone signalling. Transcription factor families, including bHLH, MYB, NAC, bZIP, C2H2, and SPL, show stage‐dependent expression. O2PLS analysis reveals covariance between metabolite and transcriptome profiles. WGCNA identifies 16 stage‐associated modules and candidate genes, including *SPL18*, *NID1*, *PIF3L6*, *ATH1L*, and *TOM6*. Alternative splicing indicates widespread post‐transcriptional regulation. Protein‐protein interaction analysis reveals interactions among AS‐O2PLS‐prioritised genes. These results show that mulching reshapes stage‐specific shoot quality formation through coordinated physiological, metabolic, transcriptional, and post‐transcriptional changes.

## Graphical abstract

Organic mulching reprograms Moso bamboo shoot development, enhancing yield and nutritional quality. This multi‐omics study reveals that coordinated metabolic, transcriptional, and post‐transcriptional regulation drives stage‐specific quality formation, offering comprehensive insights into how this agricultural practice improves crop performance and palatability.

## 1 Introduction

The global food industry increasingly demands nutritious and sustainable foods that promote human health. Meeting this demand requires eco‐friendly, cost‐effective resources that remain viable over the long term [1, 2]. Bamboo shoots, especially those of Moso bamboo (*Phyllostachys edulis*), are nutrient‐rich edible resources widely consumed in China and East Asia and increasingly marketed worldwide [3]. Bamboo shoots are rich in nutrients, including dietary fibre, proteins, amino acids, vitamins, minerals, and bioactive compounds, and possess sensory traits such as tenderness, sweetness, bitterness, stage‐dependent quality, and palatability, which strongly influence their commercial value. These quality traits change as shoots develop and reflect shifts in sugars, amino acids, phenolics, and structural cell‐wall components [3–7].

However, fluctuations in soil temperature, moisture, aeration, and nutrient availability can restrict Moso bamboo shoot production and quality [3]. In particular, low temperatures reduce shoot growth by suppressing genes involved in cell division, including *PeCDC20.1*, *PeCYC1BAT*, *PeCYCB2;4*, *PeMAD2*, *PeTCX2*, and *PeTPX2*, as well as cell elongation genes such as *PeEXPA1* [8–11]. In addition, as shoots develop and emerge, lignification and secondary cell‐wall deposition reduce tenderness and eating quality, while post‐harvest physiological changes such as xylogenesis may further compromise shoot quality. Together, these constraints hinder stable shoot production [3, 12–14].

To increase bamboo shoot production, farmers use organic mulching. This method typically uses organic materials such as manure, straw, bamboo leaves, and bran to cover the soil during winter [15]. Mulching raises soil temperature, promotes earlier shoot growth and emergence, extends the harvest window, and increases the yield of winter shoots (WS) [16]. Mulching‐treated shoots (MtS) also often show better taste and market quality, resulting in higher prices and increased economic returns for farmers. In China, particularly in Zhejiang Province, many farmers have practised mulching continuously for more than a decade because it increases yield and improves economic returns [17].

Nevertheless, our recent work and other field observations suggest that continuous mulching for more than 3 years can reduce overall bamboo forest productivity by approximately 60% [18, 19]. This decline may reflect altered soil temperature, respiration, nutrient cycling, and microbial communities, along with soil hypoxia, upward rhizome growth, and reduced rhizome vitality [17, 20–25]. These findings suggest that the benefits of mulching may decline under long‐term continuous application [26]. By contrast, single‐year mulching can increase shoot number and improve taste‐related traits and nutritional composition compared with unmulched shoots [19]. However, even short‐term mulching is labour‐intensive and costly because organic materials must be removed in summer and reapplied in winter each year. This limits the long‐term feasibility of mulching for bamboo shoot production.

Understanding how molecular mechanisms regulate shoot quality may therefore help develop more sustainable and cost‐effective production strategies. Previous studies have mostly examined rapid growth and abiotic stress responses in Moso bamboo [8, 27–34]. Our recent Viewpoint article also proposed that rapid shoot growth is driven by highly coordinated cellular, hormonal, and transcriptional programmes organized around four testable pillars [35]. Despite these advances, only one earlier study comparing WS and spring shoots (SS) in Moso bamboo suggested that dynamic metabolic and transcriptional regulation, rather than yield alone, shapes shoot quality [14]. Similarly, studies in other bamboos, such as Lei bamboo (*P. violascens*), Giant bamboo (*Dendrocalamus latiflorus*), Sweet dragon bamboo (*D. brandisii*), and Giant timber bamboo (*Bambusa oldhamii*), showed that shoot developmental stage, tissue position, metabolite composition, and environmentally regulated gene‐expression profiles also shape bamboo shoot quality [6, 36–39]. Ma et al. also reviewed the nutritional profile, storage challenges, and processing technologies of bamboo shoots [3]. However, the molecular mechanisms underlying mulching‐induced stage‐specific shoot quality formation remain poorly understood.

To address this gap, we investigated how mulching reshapes physiological traits, metabolite profiles, gene expression, and co‐expression networks across Moso bamboo shoot developmental stages. We compared WS, SS, and MtS and divided MtS into four developmental stages (MtS1‐MtS4) to capture stage‐specific molecular changes associated with quality formation. We first evaluated shoot morphology, yield, and market value, and then quantified major nutritional and quality‐related traits, including soluble sugars, proteins, fibre, soluble solids, phenolics, tannins, amino acids, antioxidant capacity, moisture content, cellulose, crude fat, and sugar‐to‐acid ratio. To uncover the molecular basis of these changes, we performed integrated metabolomic and transcriptomic analyses to identify differentially accumulated metabolites, differentially expressed genes (DEGs), enriched pathways, and co‐expression modules associated with mulching‐induced quality formation. We integrated these datasets using two‐way orthogonal partial least squares (O2PLS) and weighted gene co‐expression network analysis (WGCNA), identified stage‐specific candidate genes associated with shoot quality development, and confirmed their RNA‐seq expression patterns by quantitative real‐time PCR (qRT‐PCR). Together, our analyses provide a molecular basis for understanding stage‐specific quality development in MtS and offer a framework for developing alternative, cost‐effective strategies to improve bamboo shoot quality and production efficiency.

## 2 Materials and Methods

### 2.1 Mulching Study Sites

We conducted this study in three different Moso bamboo plantations, each located in a different bamboo forest in Huzhou City, Zhejiang Province, China: (1) Guanshan Village, Miaoxi Town, Wuxing District; (2) Yu Village, Tianhuangping Town, Anji County; and (3) Cishan Village, Meishan Town, Changxing County. These sites were abbreviated as WX, AJ, and CX, respectively, and these abbreviations are used throughout the manuscript, including in the figures. Both the WX site (120°02′ E, 30°76′ N) and the AJ site (119°36′ E, 30°31′ N) have a northern subtropical humid monsoon climate, with an annual mean temperature of 15–18°C and mean annual precipitation of 1200–1600 mm. The altitude is approximately 50 m above sea level at the WX site and 200–300 m above sea level at the AJ site. The CX site (119°57′ E, 30°39′ N) has a humid mid‐subtropical monsoon climate, with an annual mean temperature of 15.2°C, mean annual precipitation of 1400 mm, and an altitude of 78 m above sea level.

### 2.2 Mulching Experimental Design and Treatment

The experimental design and mulching treatment were based on our previous study [19], with slight modifications. In brief, because 15–20°C is considered optimal for bamboo shoot production, the experiment was conducted during two consecutive mulching seasons, from November 2019 to March 2020 and from November 2020 to March 2021. We selected Moso bamboo stands with comparable stand structure, density, age composition, and environmental conditions. At each site, nine 10 m × 10 m experimental plots were established, comprising three replicate plots for each group. A 3‐m‐wide buffer zone was maintained around each plot to minimize interference between treatments. Before the mulching treatment, in the last week of November of each season, we first applied a 3‐cm layer of organic fertilizer and watered the soil thoroughly. We then applied a 5–10‐cm layer of rice straw and lightly watered it to accelerate fermentation and heat generation in the lower layer. Next, we applied a 20‐cm layer of bamboo leaves, followed by a 20‐cm layer of bran as an insulating layer. When bamboo shoots began to emerge naturally, the mulching materials were gradually removed in the last week of March 2020 and March 2021. As mulching spans two calendar years, the 2020 season refers to the mulching cycle from November 2019 to March 2020, whereas the 2021 season refers to the cycle from November 2020 to March 2021.

### 2.3 Shoot Sample Collection for Agronomic Performance and Market Assessment

To analyse shoot morphology and biochemical traits, we examined three sample groups: WS, SS, and MtS. WS and SS developed naturally in the same Moso bamboo forests without mulching, whereas MtS developed earlier under the mulching treatment. WS and MtS were sampled in mid‐to‐late February of each season by carefully digging them out, whereas SS were sampled in mid‐to‐late April of each season. For each sample group, five biological replicates were collected and analysed immediately. To evaluate shoot production and market value, we selected 12 independent farming households involved in selling WS, SS, and MtS in their local markets and recorded the market price of each bamboo shoot type. These households were treated as independent economic replicates. Based on these records, we analysed yield, cost, output, and net profit per square hectometre (hm^2^) across the three sites (WX, AJ, and CX).

### 2.4 Shoot Sample Collection for Physicochemical, Metabolomic, and Transcriptomic Analyses

Based on the higher yield and net profit of MtS in 2020 and 2021, we repeated the mulching experiment at the CX site from November 2024 to March 2025 for physiological and molecular analyses. For stage‐specific MtS sampling, we established a 5 m × 5 m subplot within each 10 m × 10 m mulching experimental plot. After removing the mulching materials, we classified MtS into four developmental stages (MtS1‐MtS4) according to their emerged height above the soil surface. Shoots measuring approximately 5, 10, 15, and 20 cm were classified as MtS1, MtS2, MtS3, and MtS4, respectively. MtS1‐MtS4 were sampled on 19 February 2025 by carefully digging them out, whereas SS were sampled at a single stage on 17 April 2025 for comparative analysis. Mulching advanced shoot emergence; therefore, MtS and SS were collected at different times. Among the four MtS stages, MtS4 was considered developmentally comparable to SS based on shoot height. Thus, we performed comparisons between SS and MtS, as well as among the four MtS stages, for quality traits, metabolomics, and transcriptomics. WS were therefore excluded from these comparative studies. Three biological replicates from each group were collected and processed immediately. Representative tissues were then frozen in liquid nitrogen and stored at −80°C for subsequent physicochemical, metabolomic, and transcriptomic analyses.

### 2.5 Shoot Morphology and Biochemical‐Trait Measurements

Freshly collected shoot samples from all three groups were first photographed for morphological analysis and then brought to the laboratory. The shoot sheaths were removed immediately, after which the shoots (edible parts only) were sliced into long strips using a stainless‐steel knife and thoroughly mixed. The samples were then divided into four equal parts, subsampled by quartering, heated at 105°C for 30 min to inactivate enzymes, oven‐dried at 75°C, ground, passed through a 60‐mesh sieve, and stored in wide‐mouth bottles in a desiccator until further use. To compare biochemical traits among all three groups, we measured total sugars, proteins, crude fibre, and soluble solids. Specifically, total sugar content was determined using the anthrone colourimetric method [40]. Protein content was determined according to a Chinese national standard, whereas crude fibre and soluble solids contents were determined according to Chinese agricultural industry standards. Specifically, protein content was determined according to GB 5009.86–2016, Determination of Protein in Foods. Crude fibre content was determined according to NY/T 1459–2007, Determination of Crude Fibre in Vegetables: Enzymatic Gravimetric Method. Soluble solids content was determined according to NY/T 2637–2014, Determination of Soluble Solids in Fruits and Vegetables: Refractometric Method.

### 2.6 Comparative Quality‐Trait Measurements Between SS and MtS1‐MtS4

For comparative analysis between SS and MtS1‐MtS4, we measured quality‐related physicochemical traits, including crude fat, DPPH radical‐scavenging activity, amino acid content, dietary fibre, total phenolic content, moisture content, tannin content, total sugar content, total antioxidant capacity, total acid content, cellulose content, and sugar‐to‐acid ratio. These traits were analysed by Metware Biotechnology Co., Ltd., Wuhan, China (https://www.metware.cn/), using Chinese national standards, standard protocols, and commercial assay kits according to the manufacturers’ instructions. Specifically, crude fat was determined by Soxhlet extraction with petroleum ether according to GB 5009.6–2016. DPPH radical‐scavenging activity was measured using a commercial assay kit (Norminkoda Biotechnology Co., Ltd., Wuhan, China; NM‐W‐0111) at 515 nm after ethanol extraction. Amino acid content was determined using a ninhydrin‐based commercial kit (Norminkoda Biotechnology Co., Ltd., Wuhan, China; NM‐W‐0718) at 570 nm. Total dietary fibre was determined by the enzymatic‐gravimetric method according to GB 5009.88–2014 after sequential digestion with thermostable α‐amylase, protease, and amyloglucosidase. Total phenolic content and tannin content were measured colourimetrically at 760 nm. Total sugar content was determined using the 3,5‐dinitrosalicylic acid (DNS) colourimetric method at 540 nm. Total antioxidant capacity was determined using the FRAP (ferric reducing antioxidant power) assay. Moisture content was determined by the oven‐drying method to constant weight. Total acid content was measured according to GB/T 12456–2008 by acid–base titration with standard NaOH using phenolphthalein as the endpoint indicator. Cellulose content was determined by acid hydrolysis followed by anthrone‐based colourimetric quantification at 620 nm. Sugar‐to‐acid ratio was calculated from total sugar and total acid contents.

### 2.7 Extraction and Data Analysis of Shoot Metabolites

For metabolomics analysis, three biological replicates of SS and MtS1‐MtS4 were used. Metabolomic analysis was conducted by Metware Biotechnology Co., Ltd., following its standard plant metabolomics workflow. Metabolite abundance data were subjected to principal component analysis (PCA) to assess overall separation among SS and MtS1‐MtS4. Differentially accumulated metabolites were identified using VIP ≥ 1 and |log2FoldChange| ≥ 1, according to the Metware workflow. These metabolites were then used for downstream joint analyses with the transcriptome, including shared KEGG and MetMap pathway analysis, Pearson correlation analysis, canonical correlation analysis (CCA), and O2PLS [41].

### 2.8 RNA Extraction, Library Preparation, and Transcriptome Sequencing

Total RNA was extracted from representative shoot tissues using a CTAB lysis buffer and an automated magnetic‐bead purification workflow with on‐board DNase I digestion. Briefly, shoot samples (50–100 mg) were ground in liquid nitrogen, mixed with CTAB composite lysis buffer, incubated at 65°C for 10 min, centrifuged, and subjected to chloroform extraction, after which the supernatant was transferred to a 96‐well plate for automated purification. RNA concentration and integrity were assessed using a Qubit 4.0 fluorometer/MD microplate reader and a Qsep400 bioanalyser, respectively. After quality control, mRNA was isolated and reverse‐transcribed into cDNA, followed by library preparation through end repair, A‐tailing, adaptor ligation, purification, PCR amplification, denaturation, and circularization. The cDNA libraries were first quantified using Qubit, their insert size was checked using an Agilent 2100 system, and the effective library concentration was determined by qPCR (> 2 nM). Finally, DNA nanoball (DNB) preparation, chip loading, and sequencing were performed on the MGI sequencing platform by Metware Biotechnology Co., Ltd.

### 2.9 Transcriptome Data Processing and Differential Expression Analysis

Following sequencing, raw FASTQ reads were filtered to remove adapter sequences, paired reads with > 10% ambiguous bases (N), and paired reads with > 50% low‐quality bases (Q ≤ 20). Clean reads were aligned to the Moso bamboo reference genome [42] using HISAT2 with default parameters. Transcript assembly and novel transcript prediction were performed using StringTie. Gene read counts were generated using featureCounts, and transcript abundance was normalized as fragments per kilobase of transcript per million mapped reads (FPKM). PCA and sample correlation analyses were used to evaluate overall transcriptomic similarity among samples. Differential expression analysis was performed using DESeq2 [43], and genes with |log2FoldChange| ≥ 1 and false discovery rate (FDR) < 0.05 were considered DEGs.

Novel transcripts were compared with the reference annotation to identify novel genes, and their sequences were annotated against the Kyoto Encyclopedia of Genes and Genomes (KEGG), Gene Ontology (GO), NR (NCBI non‐redundant protein sequences), Swiss‐Prot, TrEMBL, and eukaryotic orthologous groups (KOG) databases using DIAMOND. Functional enrichment analyses of DEGs were then performed using hypergeometric tests. Gene set enrichment analysis (GSEA) [44] was additionally performed on ranked gene lists without imposing a predefined DEG threshold, with all expressed genes ranked by log2 fold change. This approach allowed coordinated but moderate transcriptional shifts to be detected at the pathway level. Enrichment was evaluated using the normalized enrichment score (NES), and gene sets with FDR < 0.05 were considered significantly enriched.

### 2.10 Weighted Gene Co‐Expression Network Analysis

Pearson correlation coefficients between gene pairs were calculated as part of the WGCNA workflow using the FPKM expression matrix [45]. Before the network construction, low‐expression genes and genes with little variation across samples were removed using the varFilter function in the R genefilter package. A soft‐thresholding power was selected using the pickSoftThreshold function in the WGCNA package with RsquaredCut = 0.85. Genes were then clustered into co‐expression modules based on topological overlap and hierarchical clustering. Module‐sample relationships were used to identify modules associated with SS and MtS samples, and hub genes were prioritised according to intramodular connectivity for downstream validation.

### 2.11 Integrated Metabolomic and Transcriptomic Analyses

Integrated metabolome–transcriptome analyses were performed using the conjoint analysis workflow provided by Metware Biotechnology Co., Ltd. The metabolomic and transcriptomic datasets were first subjected to PCA independently to assess sample separation among groups. Differentially accumulated metabolites and DEGs were then mapped to shared KEGG pathways. When both datasets contained MetMap annotations, shared MetMap pathways were also included in the joint pathway analysis. Metabolites and genes were simultaneously projected onto common pathway maps to identify coordinated metabolic and transcriptional shifts. Gene–metabolite associations were assessed by Pearson correlation analysis using quantitative values across all samples. Correlations with an absolute Pearson correlation coefficient > 0.8 and *p* < 0.05 were retained as significant. For differential gene–metabolite pairs, nine‐quadrant plots were used to compare log2 fold changes of genes and metabolites and to distinguish concordant and discordant regulatory patterns. Correlation heatmaps and network plots were generated to visualize positive and negative gene–metabolite associations. CCA was performed within shared pathways to evaluate the multivariate associations between differentially accumulated metabolites and DEGs. O2PLS analysis was further used to partition the metabolomic and transcriptomic datasets into joint, orthogonal, and residual components and to identify variables with the strongest cross‐omics contributions. CCA was implemented using the R package CCA, and O2PLS was implemented using OmicsPLS [46], with optimal model parameters selected by cross‐validation.

### 2.12 Alternative Splicing Analysis

Alternative splicing (AS) events were identified from RNA‐seq alignment files using rMATS [47]. Five major AS types were analysed: exon skipping (ES), alternative 5′ splice site (A5SS), alternative 3′ splice site (A3SS), mutually exclusive exons (MXE), and intron retention (IR). AS events were quantified using both junction counts (JC) and junction counts plus exon counts (JCEC). AS events with FDR < 0.05 were considered differentially spliced events. To examine whether quality‐associated regulatory genes were also regulated at the post‐transcriptional level, we compared WGCNA‐derived hub genes with DEGs showing differentially spliced events.

### 2.13 Protein‐Protein Interaction Network Analysis

To explore functional interactions among AS‐O2PLS‐prioritised genes, protein‐protein interaction (PPI) networks were constructed using the STRING database (https://string‐db.org). Protein sequences of target DEGs were aligned to reference proteins using DIAMOND [48] to identify homologous proteins present in the STRING database. Based on the best‐matching orthologs, predicted interaction information was retrieved from STRING. The resulting predicted interaction dataset was exported as tabular files, and interactions involving only AS‐O2PLS‐prioritised genes were retained based on interaction confidence scores for network construction.

### 2.14 RNA Extraction and qRT‐PCR Analysis

The RNA‐seq expression patterns of selected candidate genes, including *SPL18*, *NID1*, *PIF3L6*, *ATH1L*, and *TOM6*, identified through WGCNA connectivity analysis, were confirmed by qRT‐PCR. Total RNA was extracted from bamboo shoots using the RNAprep Pure Plant Plus Kit (TianGen Biotech Co., Ltd., China). First‐strand cDNA was synthesised using the TransScript One‐Step gDNA Removal and cDNA Synthesis SuperMix Kit (TransGen Biotech Co., Ltd., China). qRT‐PCR was performed using the ABI StepOne Plus Real‐Time PCR System (Applied Biosystems, USA) with the ChamQ Universal SYBR qPCR Master Mix Kit (Vazyme Biotech Co., Ltd., China). Each sample was analysed with three biological replicates. *PeNTB* [49] was used as the reference gene. Relative expression levels were calculated using the 2−ΔΔ*C*T method [50]. Primers were designed using Primer Premier 5 (http://www.premierbiosoft.com/) and are listed in Table S1.

### 2.15 Statistical Analysis

PCA of quality‐related physicochemical traits was performed using standardised trait values to assess overall variation among SS and MtS1‐MtS4 shoots. Loading plots were used to identify traits contributing most strongly to the principal components. Pearson correlation analysis was conducted to evaluate relationships among physicochemical traits. Statistical analyses were performed using SPSS Statistics version 28.0 [51], and figures were generated using GraphPad Prism version 8.0.2 (GraphPad Software, San Diego, CA, USA) and the online platform SRplot [53]. Data are presented as the mean ± SD from at least three biological replicates. Differences among groups were assessed by one‐way ANOVA followed by Tukey's multiple‐comparison test. Differences were considered significant at *p* < 0.05.

## 3 Results

### 3.1 Mulching Alters Shoot Development, Yield, and Quality‐Related Physiological Traits

Mulching (Figure 1B–D) significantly promoted shoot development and yield. Temperature monitoring across months revealed that shoot growth occurred earlier, especially during the mulching period from November to February (Figure 1A). Shoot diameter increased most when mulching temperatures were between 19°C and 24°C, whereas at higher temperatures (≈27°C) shoot diameter decreased. MtS also exhibited larger diameters than WS and SS (Figure 2A) at all three sites (WX, AJ, and CX). The 2020 and 2021 harvest showed that MtS produced higher total shoot yield than WS and SS, with site‐specific increases most pronounced at WX, followed by CX and AJ (Figure 1E). Morphological and biochemical analyses showed that MtS contained higher total sugar, protein, and soluble solids compared with WS and SS (Figure 2B–E), indicating altered quality‐related biochemical properties. Collectively, these data demonstrated that mulching enhanced shoot development, yield, and quality‐related biochemical traits.

**FIGURE 1:**
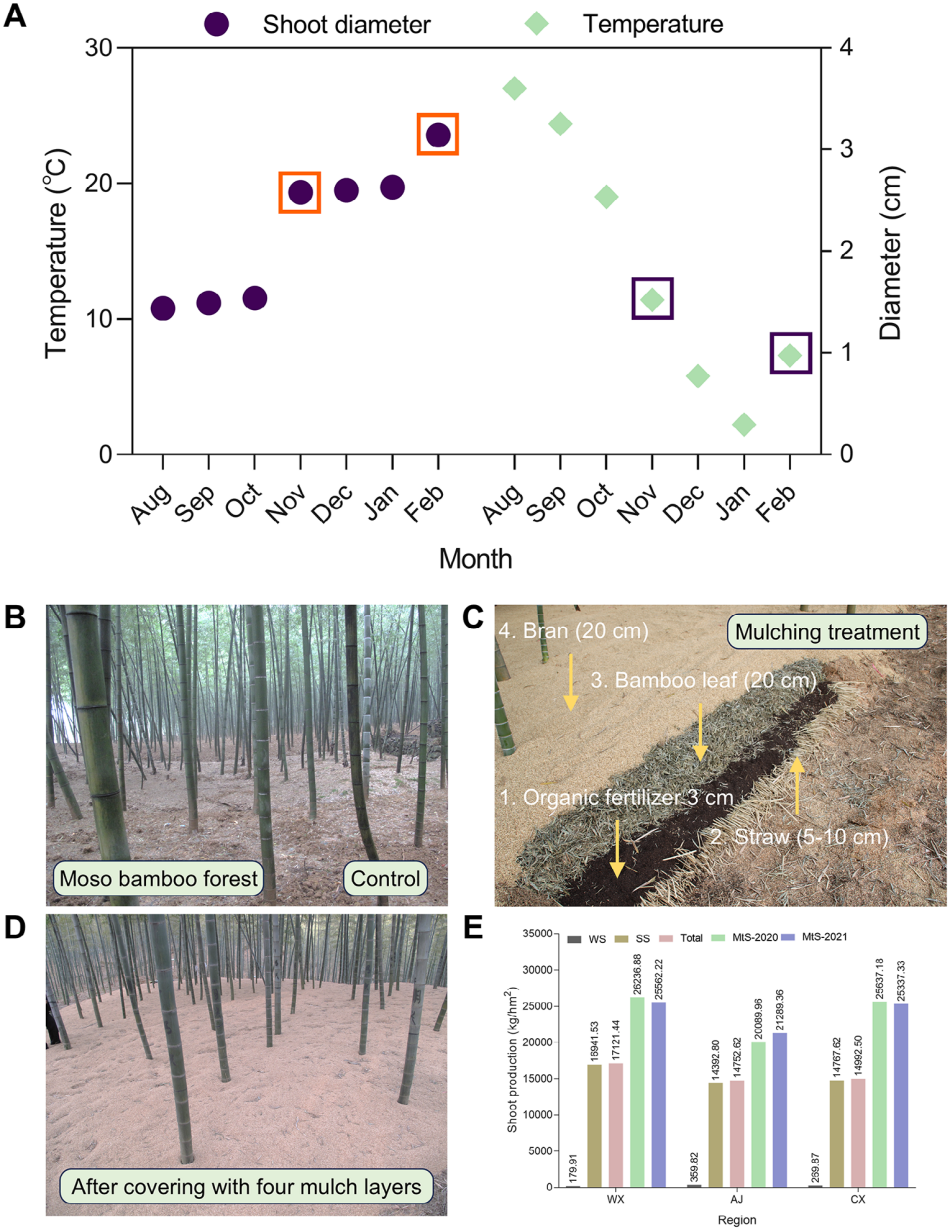
Mulching experimental design and field performance of Moso bamboo shoots. (A) Temperature changes across months show corresponding changes in shoot diameter and define the suitable period for mulching treatment, especially from November to February. Highlighted circles and diamonds indicate shoot diameter and temperature, respectively. (B) Representative control Moso bamboo forest at the CX site. (C) Mulching treatment showing the four applied layers: organic fertilizer, straw, bamboo leaves, and bran. (D) Moso bamboo forest after covering with four mulch layers. (E) Production of winter shoots (WS), spring shoots (SS), and mulching‐treated shoots (MtS) at the WX, AJ, and CX sites. The 2020 and 2021 bars represent MtS production during the November 2019 to March 2020 and November 2020 to March 2021 mulching seasons, respectively. WX represents Guanshan Village, Miaoxi Town, Wuxing District; AJ represents Yu Village, Tianhuangping Town, Anji County; and CX represents Cishan Village, Meishan Town, Changxing County.

**FIGURE 2:**
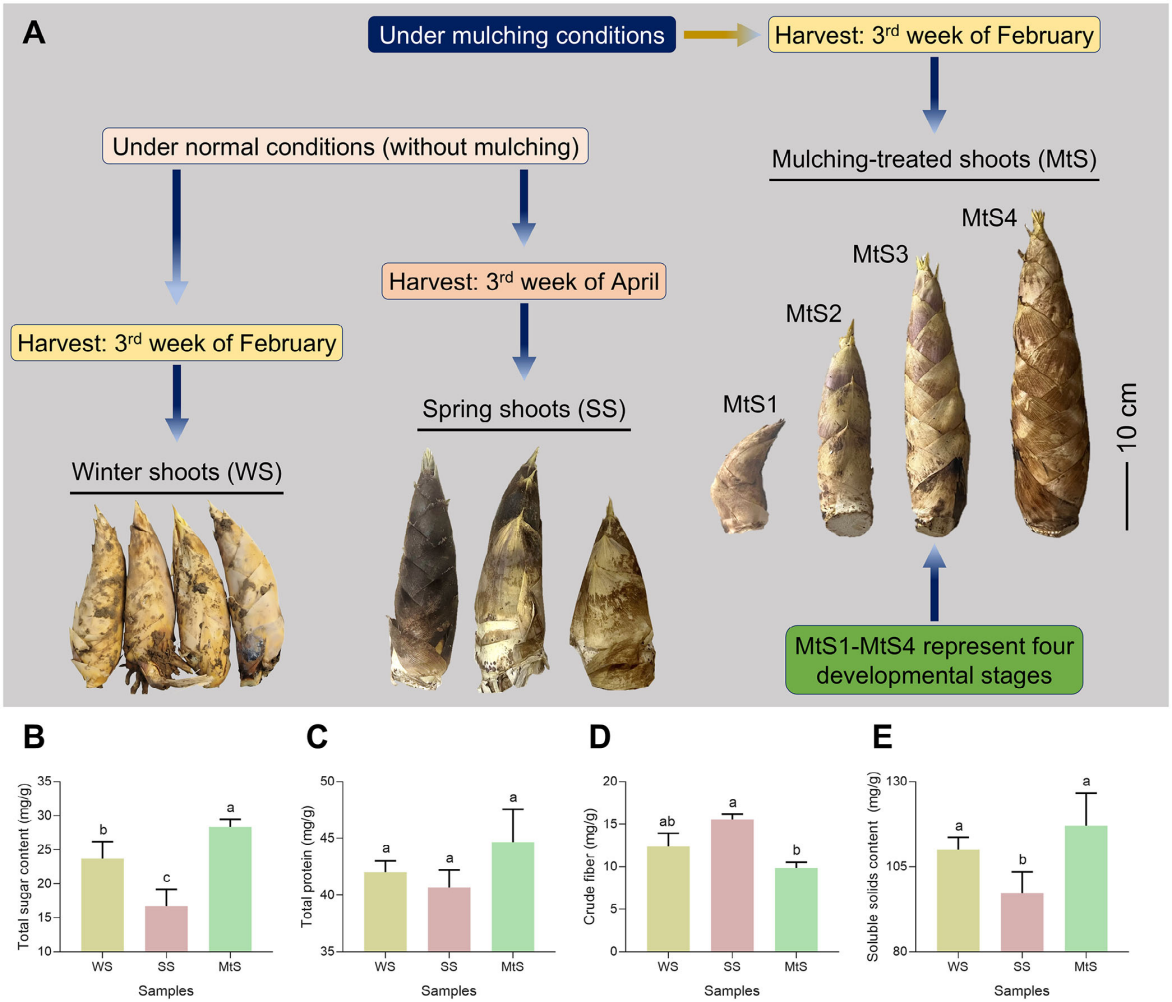
Morphological and biochemical differences among Moso bamboo winter shoots (WS), spring shoots (SS), and mulching‐treated shoots (MtS). (A) Representative photographs of WS, SS, and four developmental stages of mulching‐treated shoots (MtS1‐MtS4). Scale bar, 10 cm. (B‐E) Total sugar content, total protein content, crude fibre content, and soluble solids content in WS, SS, and MtS. Data are shown as the mean ± SD. Different letters indicate significant differences among groups (*p* < 0.05).

### 3.2 MtS Exhibits Higher Market Value and Economic Benefits

Mulching significantly increased market value and farmers’ economic returns. MtS exhibited higher and more consistent local market prices than SS, reaching approximately 9–10 CNY per kg, compared with 1–2 CNY per kg for SS (Figure 3A). Although WS occasionally reached higher prices (11–17 CNY per kg), MtS prices were more consistent due to uniform harvest timing and shoot nutritional quality. Total output and net profit analyses showed that MtS outperformed SS across all three sites: WX had the highest net profit, increasing from ∼29 130 CNY/ha for SS to ∼223 493 CNY/ha for MtS, followed by CX (from ∼22 998 to 218 380 CNY/ha) and AJ (from ∼19 775 to 179 475 CNY/ha) (Figure 3B). The 2020 and 2021 harvest indicated that 2020 produced higher total shoot yield than 2021. However, this difference is only a tentative observation, while our previous study suggests that continuous mulching may gradually reduce productivity [19]. Household‐level data from 12 farmers further confirmed these benefits: MtS exhibited higher production and total output than SS, with several households achieving 25–40% increases in total output in 2021 (Figure 3C). These findings confirmed that mulching also improved MtS market value and household net profit.

**FIGURE 3:**
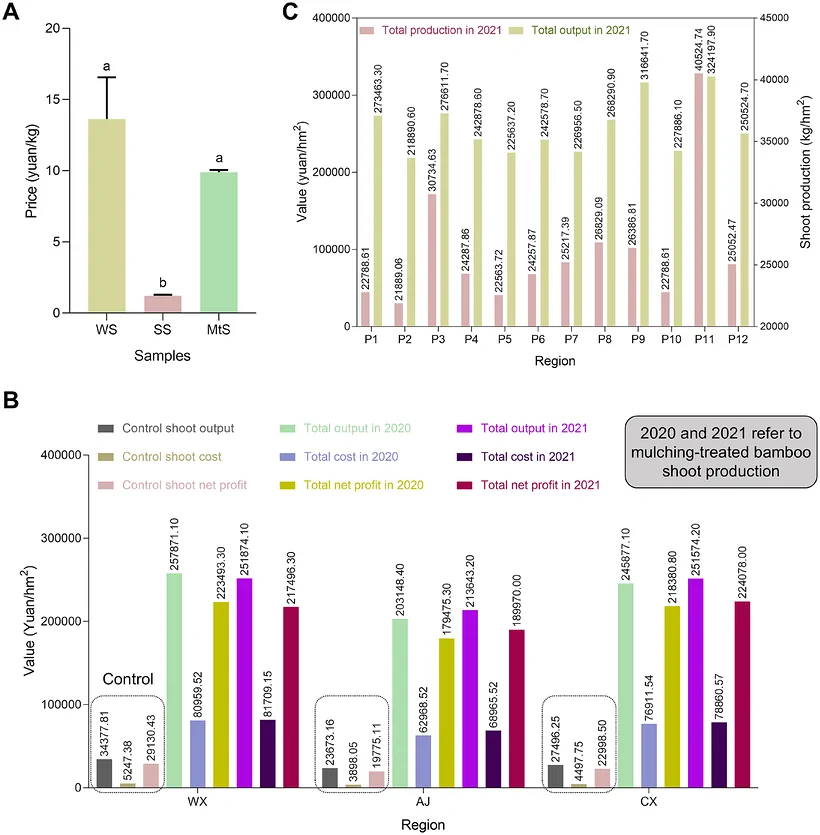
Market value, production output, and economic benefits of Moso bamboo winter shoots (WS), spring shoots (SS), and mulching‐treated shoots (MtS). (A) Local market price comparison, expressed in Chinese yuan, among WS, SS, and MtS. (B) Total output, total production cost, and net profit of SS (control shoots) and MtS at the WX, AJ, and CX sites. The 2020 and 2021 bars represent MtS production during the November 2019 to March 2020 and November 2020 to March 2021 mulching seasons, respectively. WX represents Guanshan Village, Miaoxi Town, Wuxing District; AJ represents Yu Village, Tianhuangping Town, Anji County; and CX represents Cishan Village, Meishan Town, Changxing County. (C) Household‐level shoot production and total output recorded in 2021 from 12 selected farming households (P1‐P12). Data are shown as the mean ± SD where applicable. Different letters indicate significant differences among groups (*p* < 0.05).

### 3.3 Stage‐Specific Physicochemical Changes Contribute to Shoot Quality

To investigate stage‐specific quality formation, we further divided MtS into four developmental stages (MtS1‐MtS4). Physicochemical analyses revealed stage‐dependent changes in total sugar, crude fat, amino acids, phenolics, tannin content, antioxidant capacity, cellulose content, and sugar‐to‐acid ratio (Figure 4A–L). MtS exhibited higher total sugar, total phenolic content, crude fat, cellulose content, tannin content, and sugar‐to‐acid ratio compared with SS. Antioxidant capacity, water content, and total acid content were lower in MtS, whereas DPPH radical‐scavenging activity was elevated at MtS2, and the sugar‐to‐acid ratio peaked at MtS1. PCA analysis and loading plots further revealed distinct distribution patterns among SS and MtS developmental stages (Figure 5A–D). Component 1 identified total soluble sugar, sugar‐to‐acid ratio, and total phenolic content as major contributors to sample differentiation, whereas water content, total antioxidant capacity, and total acid content showed negative contributions to PC1 (Figure 5C). Component 2 captured additional variation associated with shoot quality‐related traits (Figure 5D). Pearson correlation analysis also showed coordinated relationships among physicochemical traits. Total soluble sugar showed a strong positive correlation with the sugar‐to‐acid ratio, whereas total acid content showed an opposite relationship. Cellulose and tannin contents were positively correlated, while antioxidant‐related traits showed significant associations with phenolic‐related parameters (Figure 5E). These results suggest that stage‐associated changes in sugars, phenolics, and other physicochemical traits contribute to MtS quality formation and may be associated with improved market value.

**FIGURE 4:**
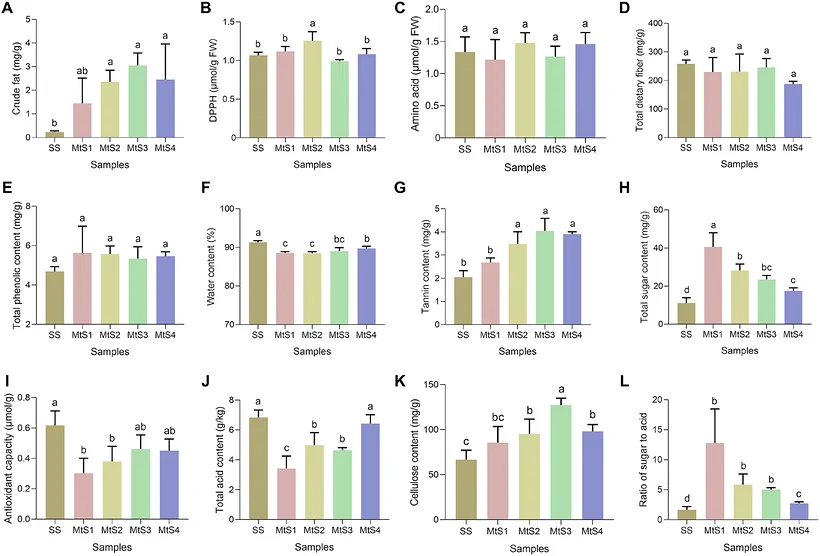
Stage‐specific physicochemical changes between Moso bamboo spring shoots (SS) and four developmental stages of mulching‐treated shoots (MtS1‐MtS4). (A‐L) Quality‐related physicochemical traits, including crude fat content, DPPH radical‐scavenging activity, amino acid content, total dietary fibre, total phenolic content, water content, tannin content, total sugar content, total antioxidant capacity, total acid content, cellulose content, and sugar‐to‐acid ratio in SS and MtS1‐MtS4. Data are shown as the mean ± SD. Different letters indicate significant differences among groups (*p* < 0.05).

**FIGURE 5:**
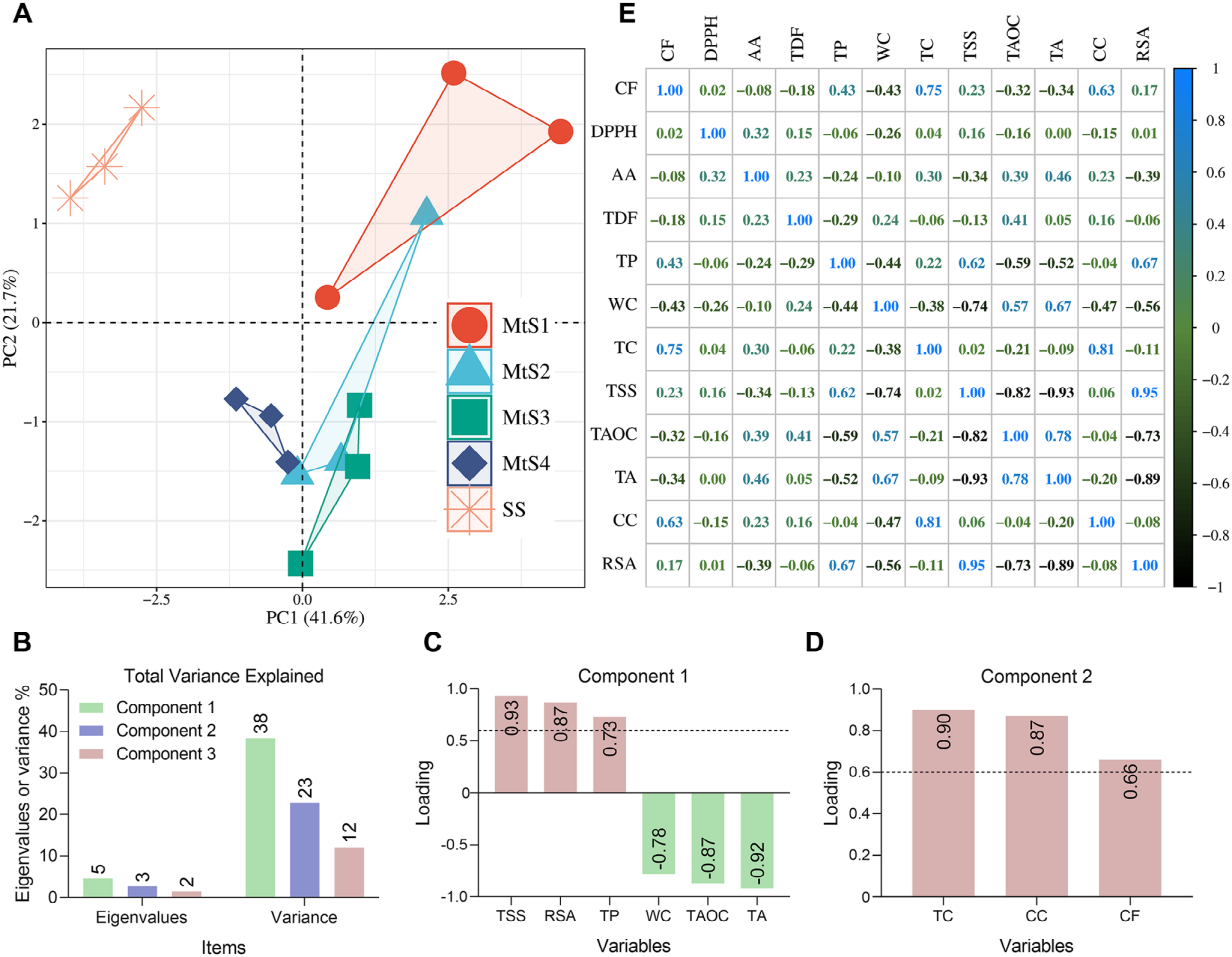
Principal component analysis (PCA) and Pearson correlation analysis among quality‐related physicochemical traits in Moso bamboo spring shoots (SS) and mulching‐treated shoots (MtS). (A) PCA score plot showing the distribution of biological replicates, with PC1 and PC2 accounting for 41.6% and 21.7% of the total variance, respectively. Distinct distribution patterns among SS and MtS stages suggest stage‐associated changes in physicochemical traits during shoot development. (B) Eigenvalues and percentage variance explained by the first three principal components. (C, D) Trait loading plots showing variables contributing to sample differentiation based on their correlation with PC1 (C) and PC2 (D). Higher absolute loading values, closer to 1.0 or −1.0, indicate stronger contributions. The dashed line indicates an approximate loading threshold of 0.6. Positive loadings for TSS, RSA, and TP indicate similar contributions to PC1, whereas negative loadings for WC, TAOC, and TA indicate opposite contributions. In PC2, TC, CC, and CF showed positive loadings, indicating their contribution to additional variation among samples. (E) Pearson correlation analysis revealed relationships among physicochemical traits across SS and MtS developmental stages. Blue indicates positive correlations, and red indicates negative correlations. Samples with high sugar and phenolic contents tend to have lower water content and acidity. AA, amino acid; CC, cellulose content; CF, crude fat content; RSA, ratio of sugar to acid; TA, total acid content; TAOC, total antioxidant capacity; TC, tannin content; TDF, total dietary fiber; TP, total phenolic content; TSS, total soluble sugar; WC, water content.

### 3.4 Metabolomic Profiling Reveals Stage‐Specific Metabolic Reprogramming

To investigate metabolic changes associated with stage‐specific shoot quality formation, we performed widely targeted metabolomic profiling across SS and MtS stages using a UPLC‐MS/MS‐based platform. In total, 2,029 metabolites were detected across all samples, covering diverse chemical classes (Table S2). Class I grouped these metabolites into amino acids and derivatives, lipids, alkaloids, terpenoids, phenolic acids, flavonoids, nucleotides and derivatives, lignans and coumarins, organic acids, and other minor classes. Among these, amino acids and derivatives represented the largest category, indicating that nitrogen‐associated metabolism is a major component of bamboo shoot metabolism. Class II further separated these metabolites into more specific subclasses, including amino acids and derivatives, phenolic acids, alkaloids, free fatty acids, sesquiterpenoids, nucleotides and derivatives, organic acids, saccharides, monoterpenoids, and LPE/LPC lipid derivatives. Flavonoid‐related subclasses, including flavones and other flavonoids, were also detected, indicating their contribution to shoot quality formation.

Subclass‐based metabolite accumulation showed distinct distribution patterns across all samples, with subclass 1 containing 718 metabolites and subclass 2 containing 589 metabolites (Figure 6A). Differences in metabolite composition between SS and MtS, with amino acids and derivatives dominating MtS and lipids dominating SS, indicated stage‐dependent metabolomic profiles (Figure 6B). Differential accumulation analysis revealed stage‐specific shifts (Tables S3–S9). SS vs MtS1 exhibited 529 upregulated and 230 downregulated metabolites. Similar trends were observed in SS vs MtS2 (477 upregulated, 273 downregulated), SS vs MtS3 (582 upregulated, 243 downregulated), and SS vs MtS4 (440 upregulated, 361 downregulated). Comparisons among MtS stages showed fewer differential metabolites. MtS2 vs MtS1 exhibited 194 upregulated and 82 downregulated metabolites; MtS3 vs MtS2 showed 100 upregulated and 221 downregulated metabolites; and MtS4 vs MtS3 showed 294 upregulated and 93 downregulated metabolites (Figure S1A–F). These results indicate that mulching induced stage‐specific metabolic reprogramming during shoot development (Figure S2A–C).

**FIGURE 6:**
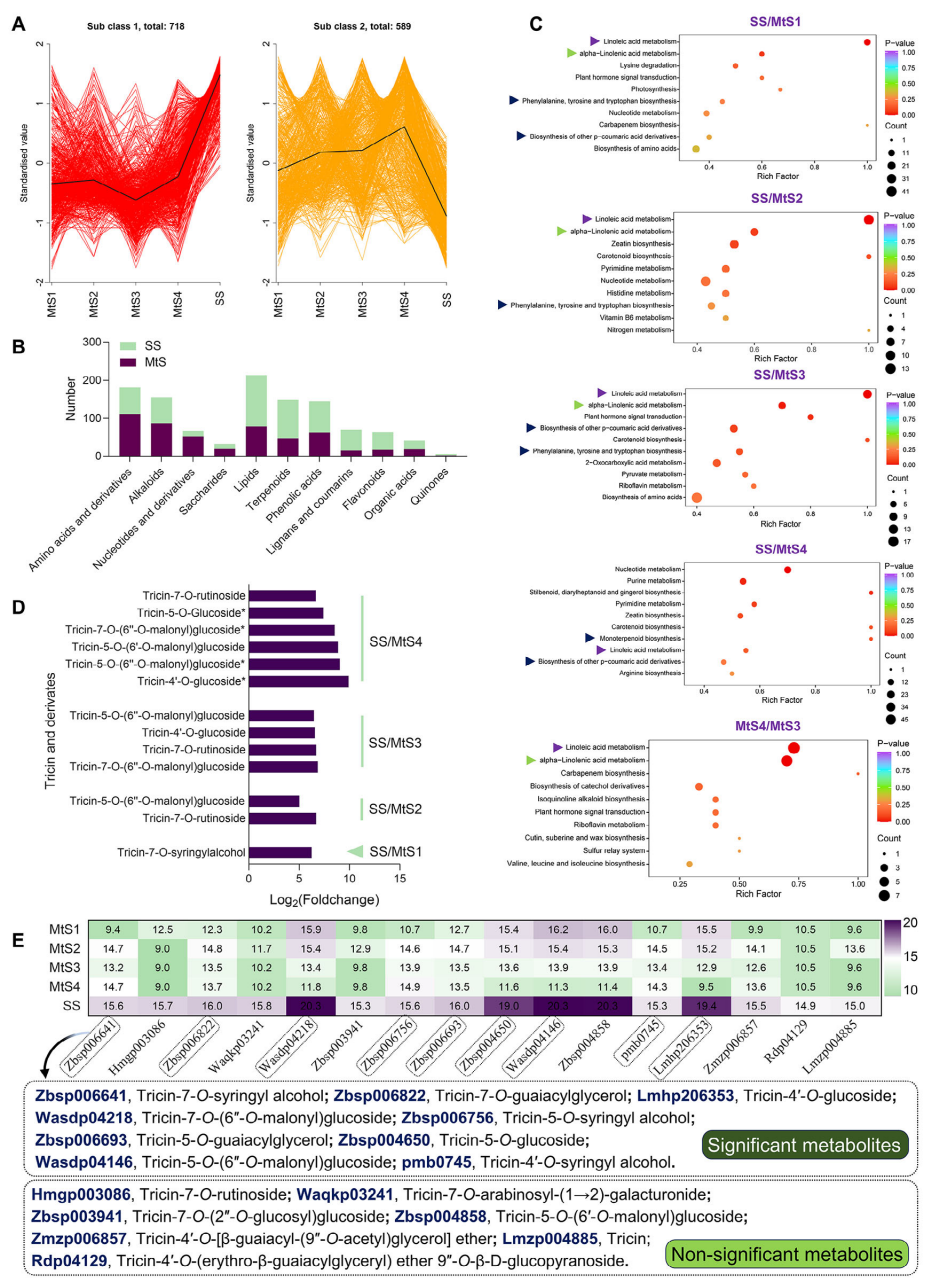
Stage‐specific metabolomic profiling between Moso bamboo spring shoots (SS) and four developmental stages of mulching‐treated shoots (MtS1‐MtS4). (A) Subclass‐based metabolite expression showing stage‐specific metabolite accumulation across SS and MtS1‐MtS4. Each thin line represents an individual metabolite accumulation pattern, and the thick line represents the average trend of the subclass across SS and MtS1‐MtS4. (B) Major classification of identified metabolites in SS and MtS. (C) KEGG enrichment of differentially accumulated metabolites in SS vs MtS1, SS vs MtS2, SS vs MtS3, SS vs MtS4, and MtS4 vs MtS3 comparisons. Bubble size indicates metabolite count, and color indicates the *p* value. Triangles indicate highlighted key metabolic pathways. (D) Differential accumulation of tricin and tricin derivatives across SS and MtS comparisons. (E) Heatmap showing the abundance of selected tricin‐related metabolites across SS and MtS1‐MtS4 comparisons. Metabolite IDs marked with rectangular outlines in the heatmap indicate significant metabolites. Full names of significant and non‐significant metabolites are listed below the heatmap.

KEGG enrichment analysis showed that amino acid metabolism, nucleotide and pyrimidine metabolism, lipid metabolism, carotenoid biosynthesis, hormone signalling, and phenylpropanoid pathways may contribute to stage‐specific quality development (Figure 6C). These pathways suggest that mulching altered metabolic and secondary‐metabolite networks associated with shoot quality formation (Tables S10–S16). Tricin and its derivatives showed clear stage‐dependent changes across SS and MtS stages (Figure 6D). Heatmap analysis of selected tricin‐related metabolites showed that several tricin derivatives were more abundant in SS and early MtS stages, whereas their abundance decreased in later MtS stages, especially MtS3 and MtS4 (Figure 6E). Of the 16 tricin derivatives analysed, nine showed significant differences and were lower in MtS compared with SS (Figure 6E), indicating that mulching strongly affected tricin‐related metabolism. Together, these results indicate that mulching induced stage‐specific metabolic reprogramming, thereby reshaping MtS‐stage‐specific metabolite accumulation and contributing to differences in quality‐related biochemical traits.

### 3.5 Transcriptomic Profiling Reveals Stage‐Specific Gene‐Expression Changes

Using RNA‐seq analysis, we next identified gene‐expression networks underlying these metabolic shifts and quality‐related traits. Across all samples, 936,623,420 clean reads were obtained, ranging from 52.37 to 71.87 million reads per sample. Of these, 915,166,665 reads were mapped to the reference genome, with an overall mapping rate of 97.71% (Table S17). PCA showed clear separation among SS and MtS samples, with PC1 and PC2 accounting for 43.40% and 15.12% of the variance, respectively. DEG analysis revealed extensive gene‐expression reprogramming between SS and MtS stages (Figure 7A).

**FIGURE 7:**
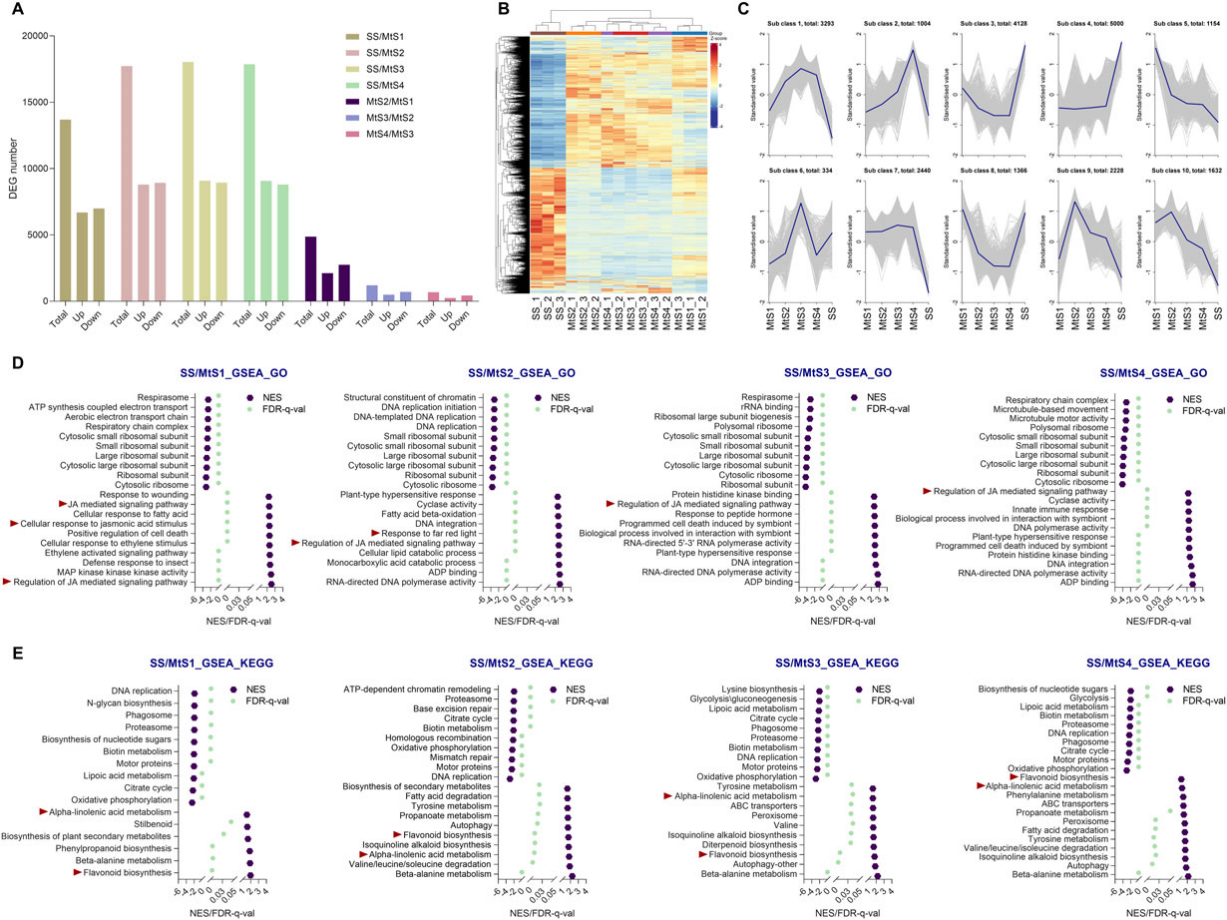
Stage‐specific transcriptomic profiling between Moso bamboo spring shoots (SS) and four developmental stages of mulching‐treated shoots (MtS1‐MtS4). (A) Number of total, upregulated, and downregulated differentially expressed genes (DEGs) in SS vs MtS and MtS stage‐to‐stage comparisons. (B) Hierarchical clustering heatmap showing global gene‐expression patterns across SS and MtS samples. Sample suffixes _1, _2, and _3 represent biological replicates. The color scale next to the heatmap indicates relative expression levels, with red representing higher expression and blue representing lower expression. (C) Subclass‐based expression patterns of clustered DEGs across SS and MtS1‐MtS4. Each thin line represents an individual gene expression pattern, and the thick line represents the average expression trend of the cluster across SS and MtS1‐MtS4. (D) Gene Ontology (GO) gene set enrichment analysis (GSEA) across SS vs MtS1, SS vs MtS2, SS vs MtS3, and SS vs MtS4 comparisons. (E) Kyoto Encyclopedia of Genes and Genomes (KEGG) GSEA of the same comparisons. Purple points indicate the normalized enrichment score (NES), and green points indicate the FDR q value.

Compared with SS, MtS1, MtS2, MtS3, and MtS4 exhibited 13,691, 17,731, 18,038, and 17,872 DEGs, respectively. These included 6,999, 8,932, 8,947, and 8,795 upregulated genes, and 6,692, 8,799, 9,091, and 9,077 downregulated genes, respectively. Stage‐to‐stage comparisons showed fewer DEGs, with 4,866 DEGs in MtS2 vs MtS1, 1,202 DEGs in MtS3 vs MtS2, and 679 DEGs in MtS4 vs MtS3 (Tables S18–S24). Hierarchical clustering and subclass‐based expression analysis further revealed stage‐dependent expression patterns (Figure 7B,C). The gene‐expression subclasses showed distinct temporal trends across SS and MtS stages, including clusters with higher expression in SS or early MtS stages and clusters with increased expression during later MtS development. Several subclasses displayed stage‐specific activation, particularly from MtS2 to MtS4. This pattern indicated distinct transcriptional programmes associated with early mulching response and later shoot quality formation. These expression patterns were consistent with the observed physiological and metabolomic differences.

Functional enrichment showed that DEGs mainly affected metabolism, development, cell‐wall pathways, secondary metabolism, and stress responses. GO and KEGG GSEA further revealed coordinated pathway enrichment related to carbohydrate and amino acid metabolism, phenylpropanoid and flavonoid biosynthesis, plant hormone signal transduction, and cell‐wall organisation (Figure 7D,E). These pathways were associated with sugar accumulation, amino acid content, antioxidant capacity, phenolic production, and fibre and cellulose accumulation during MtS development. MetMap analysis further showed that DEGs were enriched in cinnamic acid, p‐coumaric acid, flavone aglycone, sinapic acid, salicylic acid, and ferulic acid derivative pathways (Figure S3A–D). Stage‐to‐stage comparisons also showed enrichment of coumarin, kaempferol, benzoic acid, and cinnamic acid derivative biosynthesis pathways. These results suggest that phenylpropanoid‐ and flavonoid‐related transcriptional regulation may contribute to the accumulation of tricin‐related metabolites and other quality‐associated secondary metabolites in MtS.

### 3.6 Stage‐Dependent Transcription Factor Expression Patterns Across MtS Stages

We also identified 6415 differentially expressed transcription factor/transcriptional regulator (TF/TR) entries, including 5346 TF entries and 1069 TR entries, across all seven comparisons. After removing repeated Gene IDs across comparisons, these entries corresponded to 1913 unique differentially expressed TF/TR genes, comprising 1584 TFs and 329 TRs, including 68 novel TF/TR genes (Table S25). Consistent with the stage‐specific DEG patterns (Figure 8A–C), these TF/TR genes also showed stage‐dependent distribution across MtS stages (Figure 8D–F), indicating extensive transcriptional regulation during MtS development. These TF/TR genes belonged to several major families, among which bHLH, AP2/ERF‐ERF, NAC, MYB, WRKY, bZIP, and C2H2 were the most abundant, comprising 132, 117, 105, 100, 88, 82, and 79 genes, respectively (Figure 8G). These families are involved in hormone signalling, stress responses, secondary metabolism, phenylpropanoid and flavonoid biosynthesis, and cell‐wall modification. Their stage‐dependent expression may coordinate MtS developmental transitions.

**FIGURE 8:**
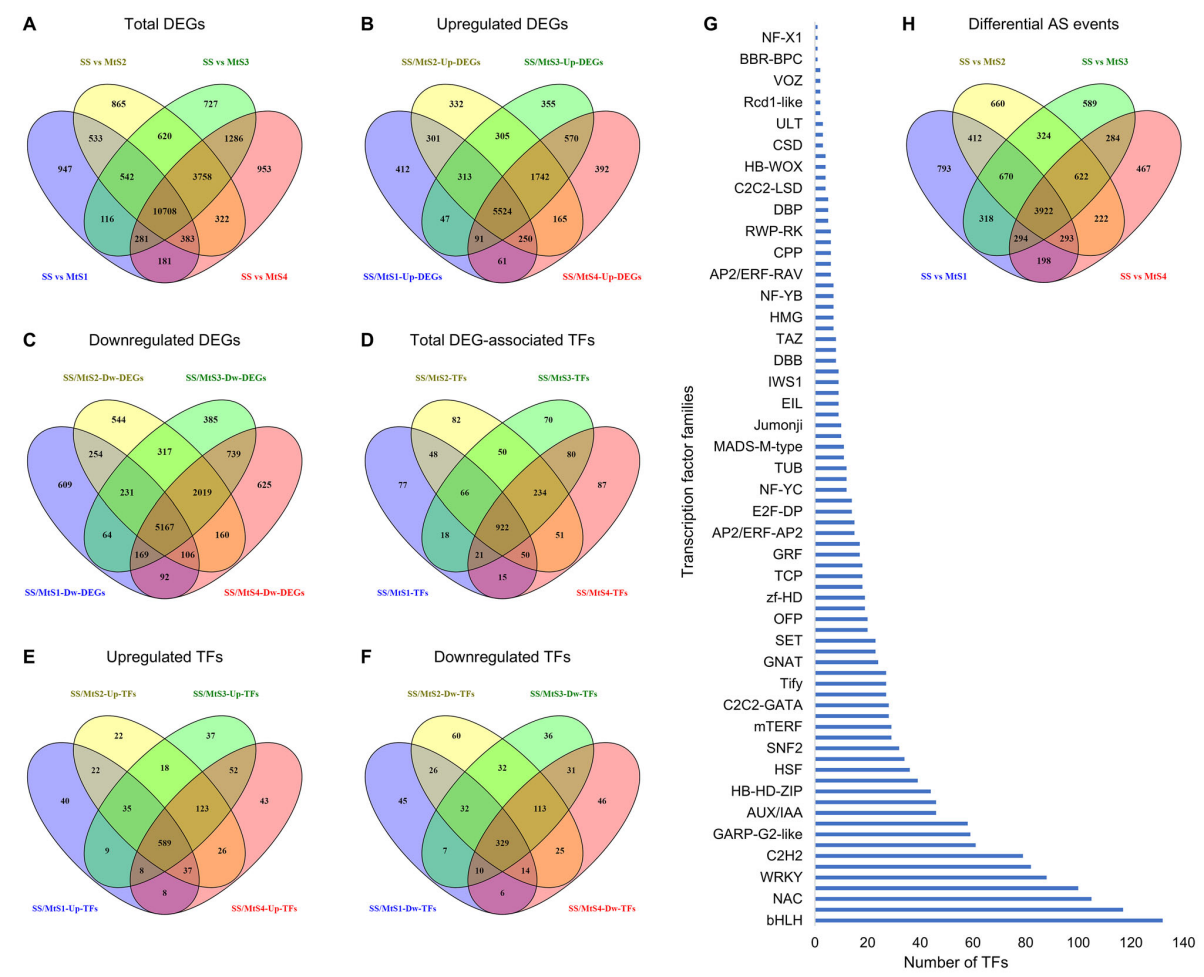
Comparative distribution of differentially expressed genes (DEGs), alternative splicing (AS) events, and transcription factors (TFs) across comparisons between Moso bamboo spring shoots (SS) and four developmental stages of mulching‐treated shoots (MtS1‐MtS4). Numbers in each Venn diagram indicate unique and overlapping genes or events among four comparisons: SS vs MtS1, SS vs MtS2, SS vs MtS3, and SS vs MtS4. (A) Total DEGs. (B) Upregulated DEGs. (C) Downregulated DEGs. (D) Total DEG‐associated TFs. (E) Upregulated TFs. (F) Downregulated TFs. (G) Distribution of DEG‐associated TFs among major transcription factor families. (H) Differential AS events.

### 3.7 Integrated Metabolomic and Transcriptomic Analyses Identify Coordinated Pathways Underlying Quality Formation

Integrated metabolomic and transcriptomic analysis based on Pearson correlation analysis, CCA, and the O2PLS model revealed strong covariance between gene expression and metabolite profiles (R^2^X = 0.83, R^2^Y = 0.92), explaining 83% of transcriptomic variation and 92% of metabolomic variation (Figure 9A). Gene loading analysis further identified the top 10 genes contributing to the shared O2PLS structure. These genes included *PH02Gene02012*, *novel.2200*, *PH02Gene40979*, *PH02Gene37115*, *PH02Gene27203*, *PH02Gene14342*, *PH02Gene08133*, *PH02Gene14451*, *PH02Gene26586*, and *PH02Gene41603*, suggesting that they are associated with mulching‐induced shoot development and quality formation (Figure 9B). For example, *PH02Gene14342*, annotated as a cycloartenol synthase‐like gene and assigned to steroid biosynthesis and metabolic pathways, suggests a possible role in sterol/triterpenoid metabolism during shoot development.

**FIGURE 9:**
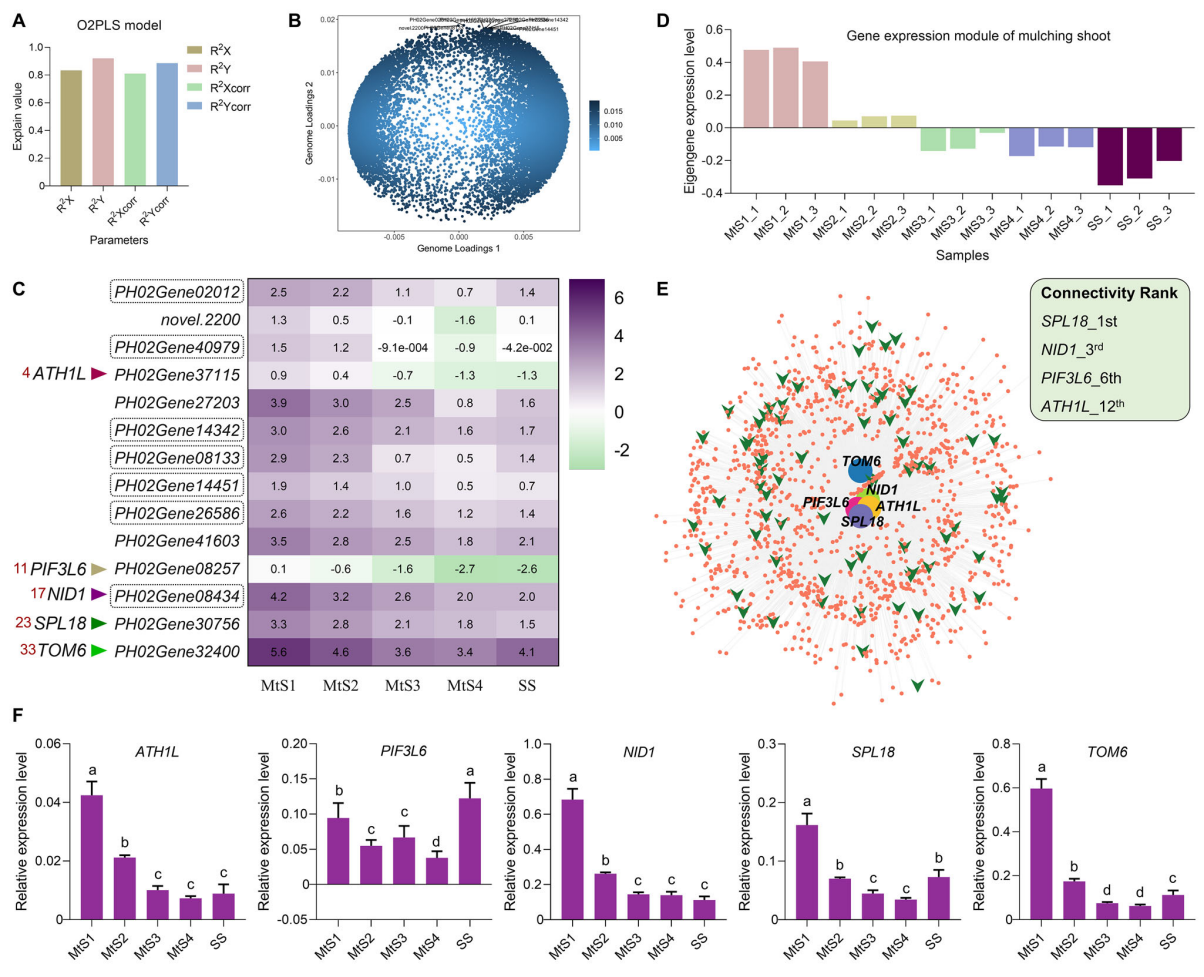
Integrated transcriptome‐metabolome analysis, WGCNA, and qRT‐PCR validation identify key candidate genes associated with shoot quality formation in Moso bamboo spring shoots (SS) and four developmental stages of mulching‐treated shoots (MtS1‐MtS4). (A) Two‐way orthogonal partial least squares (O2PLS) model parameters showing the explained variation and shared covariance between the transcriptomic and metabolomic datasets. High R^2^X and R^2^Y values indicate that the O2PLS model captured most of the variation in the respective datasets, whereas high R^2^Xcorr and R^2^Ycorr values indicate strong shared covariance between gene expression and metabolite accumulation. (B) O2PLS genome loading plot showing genes that may contribute strongly to shared transcriptome‐metabolome variation. The blue color gradient indicates the strength of gene loading, with higher values representing stronger contributions. (C) Heatmap of selected O2PLS‐prioritised genes across SS and MtS1‐MtS4, including candidate hub genes *ATH1L*, *PIF3L6*, *NID1*, *SPL18*, and *TOM6*. Gene names shown next to gene IDs were identified by NCBI BLAST searches using the coding sequence (CDS) regions of the corresponding genes. The color scale next to the heatmap indicates relative expression levels, with purple representing higher expression and green representing lower expression. Gene IDs marked in the heatmap indicate candidate genes associated with alternative splicing. (D) Eigengene expression pattern of the mulching‐associated co‐expression module across biological replicates. Positive eigengene values indicate higher module activity, whereas negative values indicate lower module activity. (E) WGCNA co‐expression network showing candidate hub genes and their connectivity ranks. (F) qRT‐PCR validation of the RNA‐seq expression patterns of *ATH1L*, *PIF3L6*, *NID1*, *SPL18*, and *TOM6* across SS and MtS1‐MtS4. Data are shown as the mean ± SD. Different letters indicate significant differences among groups (*p* < 0.05).

Similarly, the top 10 O2PLS‐associated metabolites included pmp001247, Hmhn002787, Lmmn004459, mws0847, MW0107510, MW0150989, Zbzp003271, Ymjm000139, WapTKC023606, and Wcfp003695. These metabolites comprised secondary metabolites, hormone‐related terpenoids, amino acid derivatives, nucleotide derivatives, flavonoids, lignans, and coumarins, indicating that coordinated changes in primary and secondary metabolism shape stage‐specific shoot quality formation (Figure 9C). Shared KEGG pathway analysis further highlighted coordinated regulation of flavonoid biosynthesis, phenylpropanoid metabolism, and sugar metabolism, particularly in MtS3‐MtS4 stages. Nine‐quadrant plots and Pearson correlation analysis revealed concordant regulation between tricin derivatives, phenylpropanoid‐related metabolites, and sugar metabolism‐related genes (|r| > 0.8, *p* < 0.05). Further metabolite‐gene correlation heatmaps (Figure S4A–D) showed stage‐dependent correlation patterns between key metabolites and gene expression. MtS3‐MtS4 stages exhibited the strongest coordinated clustering. These results indicate that coupled transcriptomic and metabolomic networks drive stage‐specific shoot quality formation in MtS.

### 3.8 Wgcna Identifies Stage‐Associated Modules and Candidate Hub Genes

WGCNA identified 16 co‐expression modules based on hierarchical clustering and module color assignment. The turquoise and blue modules contained 12,533 and 12,373 genes, respectively, followed by brown (2,869), yellow (2,370), green (1,244), red (434), black (429), pink (345), grey (222), magenta (187), purple (150), greenyellow (146), tan (142), salmon (90), cyan (70), and midnightblue (54) modules (Figure S5A; Table S26). Module‐sample correlation analysis revealed stage‐dependent expression patterns (Figure S5B). The blue module correlated positively with SS, whereas the turquoise and green modules correlated negatively with SS. Several modules correlated positively with MtS stages, including brown and yellow with MtS1, cyan, tan, and salmon with MtS3, and pink, red, and greenyellow with MtS4 (Table S27). Module heatmaps supported these contrasting trends across SS and MtS samples (Figure S5C): the blue module showed higher eigengene values in SS samples, whereas the turquoise and green modules showed lower eigengene values. These patterns suggest that mulching shifted major transcriptional modules, while MtS stages retained stage‐specific transcriptional signatures (Figure 9D).

The brown module contained most of the O2PLS‐prioritised genes, whereas the yellow module contained *novel.2200*. This overlap suggests that the co‐expression network also captured shared metabolite‐gene covariance. Network heatmap analysis further showed clear co‐expression blocks among selected module genes (Figure S5D). Intramodular connectivity identified candidate hub genes. In the blue module, *PH02Gene30764*, *novel.5670*, *novel.4439*, and *PH02Gene38220* ranked among the highly connected genes, while *PH02Gene19606*, an *SPL*‐related gene, also showed high intramodular connectivity, supporting its identification as a candidate hub. Annotation indicated that other blue‐module genes encode PIF‐related proteins, kinases, RNA‐processing proteins, and metabolic regulators. In the brown module, *PH02Gene07790*, *PH02Gene02174*, *PH02Gene25479*, *novel.5260*, *PH02Gene38236*, and *PH02Gene30756*, another *SPL*‐related gene, showed high connectivity (Table S26). This module also included several O2PLS‐prioritised genes, including *PH02Gene08434* (*NID1*), *PH02Gene14342*, *PH02Gene14451*, *PH02Gene27203*, *PH02Gene37115*, *PH02Gene40979*, and *PH02Gene41603*, linking brown‐module co‐expression with metabolite‐associated gene regulation. Annotation indicated that *PH02Gene37115* (*ATH1L*) encodes an ATH1‐like homeobox protein, whereas *PH02Gene08434* encodes a MYB‐related transcription factor, suggesting that developmental transcription factors may contribute to stage‐specific quality formation.

Network edge analysis revealed dense intramodular connectivity in candidate modules. In the brown module, *PH02Gene00044* connected with many co‐expressed genes and showed edge weights mostly above 0.10. Yellow and red module networks also showed strong connections among genes related to cell‐wall metabolism, xylan/xyloglucan modification, pectin metabolism, and carbohydrate pathways, highlighting cell‐wall‐associated networks in shoot quality development.

These connections suggest that hub genes act within broader co‐expression circuits associated with cell‐wall remodelling, hormone signalling, stress responses, and secondary metabolism. Within these WGCNA modules, our analysis highlighted *ATH1L*, *PIF3L6*, *NID1*, *SPL18*, and *TOM6* as candidate hub genes with high connectivity ranks, suggesting potential associations with metabolic transitions (Figure 9E). Together, the overlap among WGCNA hubs, O2PLS‐prioritised genes, and annotated developmental/metabolic genes highlights *SPL18*, *NID1*, *PIF3L6*, *ATH1L*, MYB‐related, receptor‐kinase, sulfur‐metabolism, sterol‐biosynthesis, and cell‐wall‐associated genes as candidate genes associated with bamboo shoot quality development.

### 3.9 Alternative Splicing Reveals Post‐Transcriptional Regulation During Shoot Development

To determine whether post‐transcriptional regulation contributed to mulching‐induced shoot quality formation, we analysed differential AS events between SS and MtS. Five major AS classes were detected, including ES, A5SS, A3SS, MXE, and IR. Compared with SS, MtS showed extensive AS reprogramming. Based on JCEC analysis, we identified 16,793, 17,048, 16,958, and 14,561 differentially spliced events in SS vs MtS1, SS vs MtS2, SS vs MtS3, and SS vs MtS4, respectively (Tables S28–S34). These events involved 6,900, 7,125, 7,023, and 6,302 unique AS‐associated genes, respectively (Table 1), indicating broad post‐transcriptional changes during mulching‐induced shoot development. Among the AS classes, IR, ES, and A3SS were the dominant event types, whereas MXE was consistently the least frequent. IR was the most abundant AS type in comparisons involving MtS1‐MtS3, whereas ES became dominant in SS vs MtS4, suggesting a shift in post‐transcriptional regulation at the later MtS stage. Because the comparisons were defined as SS vs MtS, increased‐inclusion events indicate higher inclusion in SS and lower inclusion in MtS, whereas decreased‐inclusion events indicate lower inclusion in SS and higher inclusion in MtS. Directional analysis showed that SS vs MtS1, SS vs MtS3, and SS vs MtS4 had more decreased‐inclusion AS events than increased‐inclusion events, indicating relatively higher inclusion in these MtS stages than in SS. By contrast, SS vs MtS2 showed slightly more increased‐inclusion events, indicating stage‐specific AS profiles in post‐transcriptional regulation during mulching‐induced shoot development (Figure 8H).

**TABLE 1:** Summary of differential alternative splicing (AS) events between Moso bamboo spring shoots (SS) and four developmental stages of mulching‐treated shoots (MtS1‐MtS4).

Comparison	Unique AS‐associated genes	Total AS events (Up/Down)	ES (Up/Down)	A5SS (Up/Down)	A3SS (Up/Down)	MXE (Up/Down)	IR (Up/Down)
SS vs MtS1	6,900	16,793 (7,571/9,222)	4,540 (2,470/2,070)	2,599 (1,482/1,117)	3,950 (1,663/2,287)	605 (282/323)	5,099 (1,674/3,425)
SS vs MtS2	7,125	17,048 (8,806/8,242)	4,587 (1,698/2,889)	2,756 (1,223/1,533)	4,228 (2,582/1,646)	610 (330/280)	4,867 (2,973/1,894)
SS vs MtS3	7,023	16,958 (8,362/8,596)	4,569 (1,549/3,020)	2,808 (1,159/1,649)	4,244 (2,476/1,768)	632 (350/282)	4,705 (2,828/1,877)
SS vs MtS4	6,302	14,561 (6,611/7,950)	4,153 (1,550/2,603)	2,424 (1,027/1,397)	3,842 (2,209/1,633)	579 (326/253)	3,563 (1,499/2,064)
MtS2 vs MtS1	2,705	4,491 (1,785/2,706)	1,375 (392/983)	615 (301/314)	855 (466/389)	173 (83/90)	1,473 (543/930)
MtS3 vs MtS2	1,340	1,843 (836/1,007)	435 (146/289)	237 (108/129)	405 (189/216)	62 (35/27)	704 (358/346)
MtS4 vs MtS3	1,815	2,663 (1,047/1,616)	561 (379/182)	280 (147/133)	481 (241/240)	67 (36/31)	1,274 (244/1,030)

*Note*: Counts are based on JCEC results (junction counts plus exon counts). Up/Down values indicate increased or decreased inclusion‐level differences according to the stated comparison direction. Unique AS‐associated genes represent non‐redundant gene IDs after removing duplicated genes across five events: exon skipping (ES), alternative 5′ splice site (A5SS), alternative 3′ splice site (A3SS), mutually exclusive exons (MXE), and intron retention (IR).

### 3.10 AS‐Associated DEGs Contribute to Mulching‐Induced Shoot Quality Formation

We next examined whether AS‐associated genes were also differentially expressed during shoot development. A total of 1994, 2878, 3097, and 2704 unique AS‐associated DEGs were identified in SS vs MtS1, SS vs MtS2, SS vs MtS3, and SS vs MtS4, respectively. In all four comparisons, AS‐associated DEGs with lower expression in MtS accounted for the larger proportion, representing 1117, 1570, 1670, and 1494 genes, respectively (Tables S35–S37). By contrast, stage‐to‐stage comparisons among MtS samples contained fewer AS‐associated DEGs, with 260, 37, and 22 genes detected in MtS2 vs MtS1, MtS3 vs MtS2, and MtS4 vs MtS3, respectively. These results suggest that transcriptional‐splicing coupling is strongest between SS and MtS stages, whereas later developmental transitions within MtS involved more restricted AS‐associated transcriptional changes. Overall, the overlap between DEGs and AS‐associated genes indicates that mulching coordinates transcriptional and post‐transcriptional processes during shoot quality development.

### 3.11 Alternative Splicing of O2PLS‐Prioritised Genes

We further compared AS‐associated genes with the top 50 O2PLS‐prioritised genes to determine whether AS also regulates genes contributing to the shared transcriptome‐metabolome structure. Among these genes, 14 showed significant AS events, comprising 55 matched AS events across MtS stages. These genes included *PH02Gene02012*, *PH02Gene03149*, *PH02Gene08133*, *PH02Gene08434* (*NID1*), *PH02Gene08563*, *PH02Gene14342*, *PH02Gene14451*, *PH02Gene26586*, *PH02Gene28040*, *PH02Gene30055*, *PH02Gene33169*, *PH02Gene40979*, *PH02Gene46544*, and *PH02Gene48480*. Among these 14 genes, *PH02Gene08563*, *PH02Gene46544*, and *PH02Gene48480* showed the largest numbers of AS events, with 13, 11, and 10 events, respectively (Tables S38 and S39). *PH02Gene28040* showed four IR events, whereas *PH02Gene03149* showed three events involving A5SS and A3SS. *PH02Gene08434* (*NID1*) showed two ES events across SS vs MtS1 and SS vs MtS2. Several genes showed only one AS event, including *PH02Gene02012*, *PH02Gene14451*, *PH02Gene26586*, and *PH02Gene30055*. This overlap suggests that stage‐dependent AS is associated with several O2PLS‐prioritised genes, including *NID1*, at the post‐transcriptional level. These events, mainly involving IR, A3SS, ES, and A5SS, may contribute to mulching‐induced shoot quality formation.

### 3.12 Protein‐Protein Interactions of AS‐O2PLS‐Prioritised Genes

We further evaluated whether the 14 AS‐O2PLS‐prioritised genes participated in protein‐protein interaction (PPI) networks. Among them, only one predicted direct interaction was detected in SS vs MtS1, between *PH02Gene26586* and *PH02Gene08133*, with a combined interaction score of 829. This suggests that these two genes may contribute to the early mulching‐induced developmental transition. The other comparisons showed no predicted direct interactions among the prioritised genes. We therefore examined the first‐degree PPI network to better understand their broader interaction context. In total, 318 first‐degree PPI edges involved at least one AS‐O2PLS‐prioritised gene across all comparisons (Tables S40–S47). SS vs MtS1 produced 303 edges and captured eight genes. In contrast, SS vs MtS2 and SS vs MtS3 produced only four and five edges, respectively, while MtS3 vs MtS2 produced six edges linked to two genes (*PH02Gene08133* and *PH02Gene30055*). SS vs MtS4, MtS2 vs MtS1, and MtS4 vs MtS3 showed no first‐degree interactions. Among these genes, *PH02Gene08563* showed the highest connectivity, forming 164 edges, followed by *PH02Gene08133* with 76 edges, *PH02Gene14342* with 35 edges, *PH02Gene26586* with 24 edges, and *PH02Gene46544* with 12 edges. In contrast, *PH02Gene14451*, *PH02Gene03149*, *PH02Gene30055*, and *PH02Gene33169* showed fewer interactions, indicating weaker network connectivity. These results suggest that these genes may influence stage‐specific shoot quality formation mainly through broader interaction neighbourhoods rather than through predicted direct interactions with each other.

### 3.13 qRT‐PCR Validates the Expression Patterns of Selected WGCNA‐Derived Candidate hub Genes

Our analysis highlighted WGCNA‐derived genes, *ATH1L*, *PIF3L6*, *NID1*, *SPL18*, and *TOM6*, as candidate hub genes associated with MtS quality formation. To validate their transcriptomic expression patterns, we further analysed these genes using qRT‐PCR. These genes showed stage‐dependent expression patterns (Figure 9F), consistent with the transcriptome, O2PLS, and WGCNA analyses (Figure 9C–E). *ATH1L*, *NID1*, *SPL18*, and *TOM6* showed higher expression in MtS1, followed by a gradual decrease across MtS2, MtS3, and MtS4, indicating their association with early shoot developmental transitions under mulching conditions. In contrast, *PIF3L6* displayed expression across SS and MtS stages, suggesting an association with later MtS developmental transitions. Collectively, the stage‐dependent expression patterns and network associations support these five genes as candidate genes associated with transcriptional and metabolic transitions during MtS development; however, their biological functions require further experimental validation.

## 4 Discussion

Low temperature restricts Moso bamboo shoot growth, whereas mulching promotes early shoot development and increases yield and economic returns. However, the molecular mechanisms underlying MtS development remain unclear. Therefore, our study aimed to clarify how mulching reshapes bamboo shoot development. Field experiments showed that mulching promoted stage‐specific shoot development and increased yield, quality‐related biochemical traits, and net profit. Compared with SS, MtS showed increased quality‐related biochemical contents, including sugar, protein, soluble solids, phenolics, cellulose, tannins, and crude fat, as well as a higher sugar‐to‐acid ratio. Metabolomic and transcriptomic analyses revealed stage‐specific shifts in amino acid, sugar, lipid, phenylpropanoid, flavonoid, and tricin‐related pathways, together with transcriptional changes associated with metabolism, hormone signalling, cell‐wall organisation, and development. Integrated metabolome‐transcriptome analysis further indicated that coupled metabolic and transcriptional programmes underlie MtS quality formation. Specifically, shoot quality formation involves coordinated transitions from primary metabolism during early development, to secondary‐metabolite accumulation during intermediate stages, and to cell‐wall‐associated structural maturation during later stages. The integrated O2PLS, WGCNA, transcription‐factor, AS, PPI, and qRT‐PCR analyses highlighted *SPL18*, *NID1*, *PIF3L6*, *ATH1L*, and *TOM6* as candidate genes associated with MtS development and quality formation. To our knowledge, this is the first study to characterise stage‐specific molecular changes in Moso bamboo.

### 4.1 Mulching Promotes Early Shoot Production, Yield, Quality‐Related Traits, and Economic Benefits

Our organic mulching significantly enhanced early shoot emergence, total yield, market value, and economic returns across all study sites (WX, AJ, and CX) compared with SS (Figures 1 and 3). These results align with previous studies showing that winter mulching increased soil temperature and modified the soil microenvironment, including organic matter content, nitrogen, phosphorus, potassium, microbial communities, and soil enzyme activities. These changes promote early shoot development and advance the harvesting period [16, 18, 24, 26]. Beyond yield analysis, our biochemical analyses further demonstrated that MtS altered quality‐related biochemical properties, including increased total sugar, protein, and soluble solids content, compared with SS and WS (Figure 2). This indicates that mulching alters metabolic activity and promotes quality‐related biochemical traits in shoots, rather than merely increasing biomass production. However, MtS showed reduced total antioxidant capacity compared with SS (Figure 4I), although some antioxidant‐related traits increased in MtS, suggesting that MtS quality is not uniform across all nutritional and functional traits. This reduction may indicate metabolic shifts from antioxidant‐related activity toward growth‐ and yield‐associated primary metabolism [3, 13, 39].

### 4.2 Stage‐Specific Metabolic Reprogramming Shapes MtS Quality and Palatability

MtS metabolic reprogramming compared with SS reflects stage‐specific coordinated pathway shifts during shoot development. An increased sugar‐to‐acid ratio in MtS indicates higher carbohydrate accumulation and lower organic acids, thereby improving sweetness and palatability. Metabolomic data suggest that mulching influences central carbon metabolism, amino acid biosynthesis, and cell wall pathways. Early‐ and mid‐MtS stages showed higher soluble sugars and amino acids, enhancing flavor‐related traits and contributing to quality‐associated biochemical changes, whereas later stages accumulated cellulose and tannin, increasing rigidity and reducing tenderness and eating quality (Figure 4). These patterns agree with previous studies showing that growth stage controls sweetness, bitterness, texture, and aroma through coordinated metabolic changes in bamboo shoots [3, 13, 39]. In *D. latiflorus*, increased cellulose and lignin at later stages promote hardening and reduce palatability [36]. From winter to spring shoots, hydrolysable tannin accumulation via gallic acid metabolism, together with lignin and cellulose biosynthesis, further reduces palatability [14]. Volatile and non‐volatile metabolites also contribute to flavor variation during shoot development, supporting stage‐dependent quality formation [7]. PCA confirmed clear stage‐wise separation of quality traits, indicating distinct metabolic profiles across developmental stages (Figure 5). These results are consistent with reports that lignification and phenolic metabolism drive quality decline during shoot maturation [14, 36].

### 4.3 Coupled Metabolomic and Transcriptomic Changes Underlie Stage‐Specific Shoot Development

Coordinated metabolic and transcriptional networks are associated with stage‐specific bamboo shoot development, physiological regulation, and quality transitions through primary and secondary metabolism, hormone signaling, and cell‐wall biosynthesis [14, 37, 52, 54]. Bamboo shoot stages also differ in amino acid, carbohydrate, and bitter‐compound metabolism [7, 39]. Similarly, our study observed stage‐dependent molecular changes. However, unlike the previous study comparing Moso bamboo winter and spring shoots [14], our results provide stage‐resolved metabolomic and transcriptomic profiling under mulching conditions. Specifically, MtS1‐MtS4 displayed distinct molecular patterns, indicating ordered metabolic and transcriptional reprogramming during MtS development (Figures 6, 7, 8, 9).

MtS1 and MtS2 showed sugar and amino acid accumulation, along with upregulation of carbohydrate metabolism and energy production genes. These changes are consistent with the high metabolic demands of rapid cell division and elongation. This pattern agrees with previous reports that early shoot development requires active primary metabolism and carbon allocation [4, 6]. In contrast, MtS3 showed increased phenylpropanoid activity and lignin precursor accumulation, together with upregulation of lignin, cellulose, and hemicellulose biosynthetic genes. This pattern aligns with earlier studies reporting a transition toward secondary metabolism during structural differentiation, including cell‐wall formation and lignification [14, 54]. MtS4 exhibited enhanced structural metabolism, higher cellulose and tannin levels, and reduced sugars and amino acids, along with upregulation of several TF families, including MYB, NAC, and SPL, suggesting a transition toward tissue maturation and lignification [54, 55]. However, the stronger stage‐dependent metabolic and transcriptional shifts compared with earlier studies suggest that mulching‐associated soil environmental changes may influence energy metabolism and carbon allocation during rapid shoot development.

### 4.4 Alternative Splicing is Associated With Stage‐Specific Shoot Development

AS is widespread in Moso bamboo. An AS atlas identified 266,711 AS events from 25,225 AS genes across 26 tissues [42]. AS regulates key Moso bamboo traits, including *PeCOL13*‐mediated flowering regulation [56], *PeGA20ox1*‐mediated GA/ABA balance and internode development [57], and growth‐related AS in dwarf mutants [58]. However, no study has reported stage‐specific isoforms regulating MtS quality formation. We detected AS‐associated DEGs across all comparisons. SS vs MtS3 showed the highest number (3,097), whereas MtS4 vs MtS3 showed the lowest number (22), indicating stronger AS regulation between SS and MtS than among MtS stages (Tables S35–S37). Among the five AS types, IR dominated shoot development, consistent with previous studies [42, 58]. These AS events may alter transcripts involved in carbohydrate metabolism, amino acid accumulation, phenylpropanoid biosynthesis, and cell‐wall development. AS‐overlapped O2PLS‐prioritised genes also linked splicing with transcriptome‐metabolome coordination. Among the 50 O2PLS‐prioritised genes, 14 showed AS events, including *PH02Gene08434* (*NID1*), which showed ES events in SS vs MtS1 and SS vs MtS2, suggesting an association between AS events and early MtS development. Other prioritised genes, including *PH02Gene08563*, *PH02Gene46544*, and *PH02Gene48480*, showed multiple AS events. Overall, our analysis identifies stage‐associated AS patterns and candidate AS events associated with MtS development. These findings extend the known roles of AS in Moso bamboo from flowering and culm development to edible shoot quality formation.

### 4.5 Predicted Protein‐Protein Interactions may Contribute to Early Mulching Responses

In Moso bamboo, PPI studies identified candidate hub genes, including *PeKNOX* genes linked with AUX/IAA signalling and rapid shoot growth [59], *ZIP* genes linked with ion transport and shoot growth [60], and hub modules linked with cell‐wall biosynthesis [61]. We detected 318 first‐degree PPI edges involving AS‐O2PLS‐prioritised genes. SS vs MtS1 showed the strongest network, with 303 edges and eight connected genes, whereas other comparisons showed fewer than six edges or no edges (Tables S40–S47). These results indicate that PPI‐based interaction patterns are broadly associated with early mulching responses. Among connected genes, *PH02Gene08563* showed the highest connectivity, followed by *PH02Gene08133*, *PH02Gene14342*, *PH02Gene26586*, and *PH02Gene46544*. These genes may be associated with metabolism, signalling, and cell‐wall‐related pathways. Overall, the predicted PPI analysis indicates a broader interaction context associated with early MtS development and links O2PLS‐prioritised genes with AS‐ and WGCNA‐supported networks.

### 4.6 Comparison of MtS Responses With Mulching‐Associated Environmental Pathways

As mulching raises soil temperature [16], we compared MtS patterns with established temperature‐ and light‐responsive pathways reported in *Arabidopsis* and rice (Table S48). MtS showed stage‐dependent changes in TFs, hormone signalling, carbohydrate metabolism, secondary metabolism, and AS events. Several changes broadly resembled environmental‐response networks reported in these model plants. *PIF3L6* also showed stage‐dependent expression, linking MtS development with *PIF*‐associated environmental signalling. However, our analyses did not identify strong HSF‐HSP or ER protein‐processing signatures associated with heat stress. Our analyses also did not highlight the strong photosynthetic and light‐harvesting responses reported under low light in rice. These comparisons provide biological context but do not establish temperature or reduced light as direct drivers of MtS responses.

### 4.7 Long‐Term Mulching Affects the Soil Microenvironment and Sustainability

Although mulching is beneficial, our previous study on Moso bamboo reported that continuous mulching (> 1 year) led to soil acidification and hypoxic conditions and altered soil pH, microbial biomass, nutrient balance, and enzyme activities, ultimately threatening long‐term bamboo forest sustainability whereas short‐term mulching increased microbial biomass and promoted bacterial activity [19]. This suggests that mulching provides short‐term yield gains but may cause long‐term soil ecological problems. Studies on Lei bamboo also showed that soil hypoxia under intensive mulching inhibited root and rhizome growth, whereas aeration improved root and rhizome development [24]. Therefore, one‐year mulching followed by one‐year recovery can maintain both productivity and soil health [19]. However, rotational mulching limits its practical adoption due to its high cost. Understanding MtS molecular mechanisms, further, could improve mulching feasibility and support sustainable bamboo culm production beyond shoot yield alone.

### 4.8 Limitations and Future Perspectives

Although our study provided multi‐omics insights into MtS development, several limitations remain. First, this study identified several candidate genes, but it did not functionally validate them because Moso bamboo lacks a stable genetic transformation system [32]. Although heterologous systems such as *Arabidopsis* can provide preliminary evidence, they cannot fully represent MtS development because *Arabidopsis* lacks underground shoot development, primary thickening growth, and pith cavity formation. Therefore, future studies should validate these genes in Moso bamboo or closely related bamboo systems using bamboo mosaic virus‐based transient expression, CRISPR ribonucleoprotein‐mediated editing, or other CRISPR‐based approaches [62–64]. Second, mulching‐induced cellular processes may differ from natural aboveground shoot development. Future functional studies should compare gene expression and metabolite accumulation under dark and light conditions. Such comparisons could clarify how light, temperature, developmental stage, seasonal environmental conditions, and their combined effects contribute to MtS quality formation. Third, this study did not examine Moso bamboo's underground architectural blueprint, such as primary thickening growth. This blueprint predetermines internode number, culm diameter, wall thickness, and pith cavity formation before shoot emergence, thereby influencing Moso bamboo rapid growth [35, 65]. Future mulching studies should integrate multi‐omics with shoot anatomical analyses, including primary thickening growth, pith cavity formation, and culm neck development, to determine whether mulching primarily promotes shoot development and quality‐related traits or also influences the underground architectural blueprint.

## 5 Conclusions

Our study revealed stage‐specific molecular dynamics (MtS1‐MtS4) associated with MtS quality formation in Moso bamboo after an initial field comparison among WS, SS, and MtS. Mulching reshaped developmental trajectories, quality‐related biochemical traits, yield, and market value through stage‐specific transcriptional and metabolic programmes (Figure 10). Multi‐omics integration revealed transcriptome‐metabolome coordination across SS and each MtS stage, supported by O2PLS covariance. Our integrated analysis highlights three major biological processes: (i) early developmental stages are characterised by carbohydrate and amino acid metabolism associated with quality‐related traits; (ii) subsequent stages involve phenylpropanoid, flavonoid, and tricin‐related secondary metabolism; and (iii) later stages involve cell‐wall biosynthesis and remodelling associated with structural maturation (Figure 10). Several TF families, including bHLH, MYB, NAC, bZIP, C2H2, and SPL, showed stage‐specific expression shifts. WGCNA identified stage‐associated modules and candidate hub genes associated with quality traits. AS‐associated DEGs indicated post‐transcriptional changes and expanded transcript diversity during shoot development. Predicted PPI analysis of AS‐O2PLS‐prioritised genes revealed a broader potential interaction context in early MtS responses compared with later stages. Together, these multi‐omics results identify candidate genes, including *SPL18, NID1, PIF3L6, ATH1L*, and *TOM6*, associated with stage‐specific MtS development and quality formation and provide targets for future functional validation.

**FIGURE 10:**
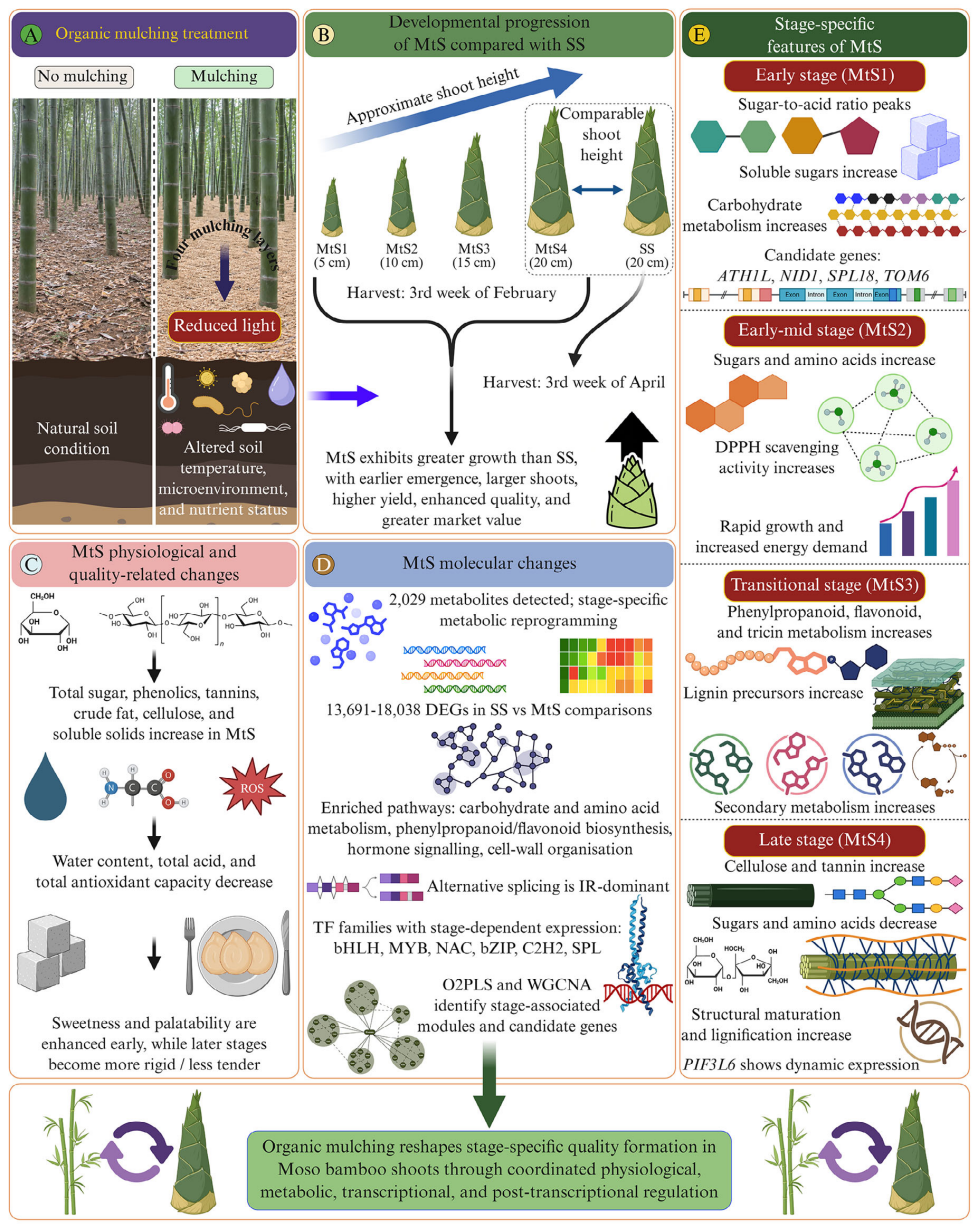
Summary of organic mulching‐associated stage‐specific quality formation in Moso bamboo shoots. (A) Schematic comparison of non‐mulched and mulched conditions, showing changes in soil temperature, microenvironment, nutrient status, and light conditions [16, 18, 24, 26]. (B) Developmental progression of mulching‐treated shoots (MtS) compared with spring shoots (SS). MtS1‐MtS4 represent approximate shoot heights of 5, 10, 15, and 20 cm, respectively; MtS4 and SS were comparable in height. (C) Major physiological and quality‐related changes in MtS, including increases in total sugar, phenolics, tannins, crude fat, cellulose, and soluble solids, together with decreases in water content, total acid, and total antioxidant capacity. (D) Major molecular changes associated with MtS development, including stage‐specific metabolic reprogramming, differential gene expression, enriched metabolic and signalling pathways, stage‐dependent transcription factor expression, intron‐retention‐dominant alternative splicing, and O2PLS‐ and WGCNA‐based identification of stage‐associated modules and candidate genes. (E) Stage‐specific features of MtS1‐MtS4, highlighting shifts in primary metabolism, secondary metabolism, cell‐wall maturation, and candidate‐gene expression. Overall, organic mulching reshapes stage‐specific quality formation through coordinated physiological, metabolic, transcriptional, and post‐transcriptional regulation. Created in BioRender. Ramakrishnan, M. (2026) https://BioRender.com/ynffeem.

## Author Contributions

B.W., H.N., Q.W., and M.R. conceived and designed the research. B.W. and Y.W. performed the mulching experiments. B.W., Y.W., C.W., P.T., Z.A., H.N., and M.R. contributed to the mulching experiments, molecular methodology, validation, formal analysis, investigation, and data curation. B.W., H.N., Q.W., and M.R. performed software analysis and visualization. B.W., Z.A., H.N., Q.W., and M.R. contributed to manuscript review and editing. Q.W. and M.R. prepared the original draft. H.N., Q.W., and M.R. supervised the study and acquired funding. Q.W., H.N., and M.R. provided resources, project administration, and overall supervision. All authors discussed the results, commented on the manuscript, and approved the final version of the manuscript.

## Declaration of Generative AI and AI‐Assisted Technologies in the Writing Process

We acknowledge that during the preparation of this work, the authors used AI‐assisted tools solely to improve grammatical clarity and enhance readability. The authors affirm that they take full responsibility for the content and scientific integrity of the publication.

## Conflicts of Interest

The authors declare no conflicts of interest.

## Supporting information




**Supporting File 1**: advs78312‐sup‐0001‐SuppMat.docx.

**Supporting File 2**: advs78312‐sup‐0002‐SuppMat.zip.

## Data Availability

The data that support the findings of this study are available from the corresponding author upon reasonable request.
