# Supplementary material for: Multi‐Omics Analyses Identify Candidate Genes Associated With Stage‐Specific Quality Formation in Mulching‐Treated Moso Bamboo Shoots
Source: Adv Sci (Weinh). 2026 Oct 30:e78312. Online ahead of print. doi: 10.1002/advs.78312 (PMC13721032; doi:10.1002/advs.78312)
Supplement: Supplementary file 1 [file ADVS-9999-e78312-s001.docx]

*Supplementary Figures*

**Multi-omics analyses identify candidate genes associated with stage-specific quality formation in mulching-treated Moso bamboo shoots**

Bo Wang ^1, 3^, Yanbin Wang ^1^, Chunyue Wang ^2^, Zishan Ahmad ^2^, Peixin Teng ^2^, Huijing Ni ^1, 3,^ *, Qiang Wei ^2,^ *****, Muthusamy Ramakrishnan ^2,^ *

^1^ Zhejiang Academy of Forestry, Hangzhou 310023, Zhejiang, China

^2^ State Key Laboratory of Tree Genetics and Breeding, Co-Innovation Center for Sustainable Forestry in Southern China, Bamboo Research Institute, School of Life Sciences, Nanjing Forestry University, Nanjing 210037, Jiangsu, China

^3^ Northwest Zhejiang Bamboo Forest Ecosystem Positioning Observation and Research Station, National Forestry and Grassland Administration, Hangzhou 310023, Zhejiang, China

* Corresponding authors’ emails: [hjni0529@163.com](mailto:hjni0529@163.com) (Huijing Ni); [weiqiang@njfu.edu.cn](mailto:weiqiang@njfu.edu.cn) (Qiang Wei); [ramky@njfu.edu.cn](mailto:ramky@njfu.edu.cn) (Muthusamy Ramakrishnan)

The following Supplementary Figures for this article are available in this file.

**Figure S1**. Differential metabolite distribution based on fold change and VIP values.

**Figure S2**. Shared and stage-specific differentially accumulated metabolites between spring shoots (SS) and the four developmental stages of mulching-treated shoots (MtS1-MtS4).

**Figure S3**. MetMap enrichment analysis of differentially expressed genes (DEGs) between spring shoots (SS) and the four developmental stages of mulching-treated shoots (MtS1-MtS4).

**Figure S4**. Stage-dependent metabolite-gene correlation networks in comparisons between spring shoots (SS) and the four developmental stages of mulching-treated shoots (MtS1-MtS4).

**Figure S5**. WGCNA module identification and module-sample relationships among spring shoots (SS) and the four developmental stages of mulching-treated shoots (MtS1-MtS4).

**Supplementary Figure S1**. Differential metabolite distribution based on fold change and VIP values. Panels A-F show pairwise comparison plots of differentially accumulated metabolites between spring shoots (SS) and the four developmental stages of mulching-treated shoots (MtS1-MtS4). The x-axis represents log2(fold change), and the y-axis represents the variable importance in projection (VIP) score. Red dots indicate significantly upregulated metabolites, green dots indicate significantly downregulated metabolites, and grey dots indicate non-significant metabolites. The dashed vertical lines represent fold-change thresholds, and the dashed horizontal line represents the VIP threshold of 1.0.

**Supplementary Figure S2**. Shared and stage-specific differentially accumulated metabolites between spring shoots (SS) and the four developmental stages of mulching-treated shoots (MtS1-MtS4). Venn diagrams show total (A), upregulated (B), and downregulated (C) metabolites across SS vs MtS1-MtS4 comparisons.

**Supplementary Figure S3**. MetMap enrichment analysis of differentially expressed genes (DEGs) between spring shoots (SS) and the four developmental stages of mulching-treated shoots (MtS1-MtS4). Bar plots show enriched MetMap pathways among DEGs in pairwise comparisons between SS and MtS stages: (A) SS vs MtS1, (B) SS vs MtS2, (C) SS vs MtS3, and (D) SS vs MtS4. The x-axis represents the percentage of DEGs assigned to each MetMap pathway, and the number beside each bar indicates the corresponding gene count and percentage.

**Supplementary Figure S4.** **Stage-dependent metabolite-gene correlation networks in comparisons** between spring shoots (SS) and the four developmental stages of mulching-treated shoots (MtS1-MtS4). Panels A–D show heatmaps of Pearson correlation coefficients between differentially accumulated metabolites and differentially expressed genes in pairwise comparisons between SS and MtS1-MtS4. The comparisons are: (A) SS vs MtS1, (B) SS vs MtS2, (C) SS vs MtS3, and (D) SS vs MtS4. Red indicates positive correlations, whereas blue indicates negative correlations. The annotation labelled “Type” distinguishes genes from metabolites, and “Class I” indicates metabolite classes.

**Supplementary Figure S5.** **WGCNA module identification and module-sample relationships among spring shoots (SS) and the four developmental stages of mulching-treated shoots (MtS1-MtS4). (A)** The h**ierarchical clustering dendrogram shows co-expressed genes and module color assignments. Each color represents one co-expression module. (B) The module-sample correlation heatmap shows associations between module eigengenes and samples from SS and MtS stages. Red indicates positive correlation, blue indicates negative correlation, and values in parentheses indicate P values. (C) The gene expression heatmap shows module expression patterns across SS and MtS samples. Red indicates higher expression, whereas blue indicates lower expression. (D) The network heatmap plot shows co-expression relationships among selected module genes. Darker regions indicate stronger intramodular connectivity and co-expression blocks.**
