# Supplementary material for: Multi‐Omics Analyses Identify Candidate Genes Associated With Stage‐Specific Quality Formation in Mulching‐Treated Moso Bamboo Shoots
Source: Adv Sci (Weinh). 2026 Oct 30:e78312. Online ahead of print. doi: 10.1002/advs.78312 (PMC13721032; doi:10.1002/advs.78312)
Supplement: Supplementary file 2 [file ADVS-9999-e78312-s002.zip › advs78312-sup-0002-00018-SuppMat/advs78312-sup-00015-S35-S37. Summary of alternative splicing-associated DEGs.docx]

*Supplementary Tables*

**Multi-omics analyses identify candidate genes associated with stage-specific quality formation in mulching-treated Moso bamboo shoots**

Bo Wang ^1, 3^, Yanbin Wang ^1^, Chunyue Wang ^2^, Zishan Ahmad ^2^, Peixin Teng ^2^, Huijing Ni ^1, 3,^ *, Qiang Wei ^2,^ *****, Muthusamy Ramakrishnan ^2,^ *

^1^ Zhejiang Academy of Forestry, Hangzhou 310023, Zhejiang, China

^2^ State Key Laboratory of Tree Genetics and Breeding, Co-Innovation Center for Sustainable Forestry in Southern China, Bamboo Research Institute, School of Life Sciences, Nanjing Forestry University, Nanjing 210037, Jiangsu, China

^3^ Northwest Zhejiang Bamboo Forest Ecosystem Positioning Observation and Research Station, National Forestry and Grassland Administration, Hangzhou 310023, Zhejiang, China

* Corresponding authors’ emails: [hjni0529@163.com](mailto:hjni0529@163.com) (Huijing Ni); [weiqiang@njfu.edu.cn](mailto:weiqiang@njfu.edu.cn) (Qiang Wei); [ramky@njfu.edu.cn](mailto:ramky@njfu.edu.cn) (Muthusamy Ramakrishnan)

The following Supplementary Tables for this article are available in this file.

**Table S35**. Summary of alternative splicing-associated differentially expressed genes during mulching-induced shoot development.

**Table S36**. Distribution of alternative splicing-associated differentially expressed genes across different alternative splicing event types.

**Table S37**. Summary of alternative splicing events overlapping with differentially expressed genes across comparisons.

**Table S35**. Summary of alternative splicing-associated differentially expressed genes during mulching-induced shoot development.

| **Comparison** | **Unique AS-associated genes** | **Unique DEG-associated genes** | **Total unique AS-DEGs** | **Upregulated AS-DEGs** | **Downregulated AS-DEGs** | **Total AS events in DEGs** |
| --- | --- | --- | --- | --- | --- | --- |
| SS vs MtS1 | 6,900 | 13,691 | 1,994 | 1,117 | 877 | 4,844 |
| SS vs MtS2 | 7,125 | 17,731 | 2,878 | 1,570 | 1,308 | 6,820 |
| SS vs MtS3 | 7,023 | 18,038 | 3,097 | 1,670 | 1,427 | 7,456 |
| SS vs MtS4 | 6,302 | 17,872 | 2,704 | 1,494 | 1,210 | 6,296 |
| MtS2 vs MtS1 | 2,705 | 4,866 | 260 | 130 | 130 | 442 |
| MtS3 vs MtS2 | 1,340 | 1,202 | 37 | 17 | 20 | 51 |
| MtS4 vs MtS3 | 1,815 | 679 | 22 | 11 | 11 | 58 |

**Note:** AS-DEGs represent genes that were both differentially spliced and differentially expressed. Upregulated and downregulated AS-DEGs follow the stated comparison direction. For SS vs MtS comparisons, upregulated AS-DEGs indicate genes with higher expression in SS and therefore lower expression in MtS.

**Table S36**. Distribution of alternative splicing-associated differentially expressed genes across different alternative splicing event types.

| **Comparison** | **AS type** | **Unique AS genes** | **Unique AS-DEGs** | **Upregulated AS-DEGs** | **Downregulated AS-DEGs** | **Dominant DEG direction** |
| --- | --- | --- | --- | --- | --- | --- |
| SS_vs_MtS1 | ES | 2262 | 613 | 380 | 233 | Up |
| SS_vs_MtS1 | A5SS | 1876 | 568 | 354 | 214 | Up |
| SS_vs_MtS1 | A3SS | 2688 | 777 | 471 | 306 | Up |
| SS_vs_MtS1 | MXE | 274 | 68 | 48 | 20 | Up |
| SS_vs_MtS1 | IR | 3418 | 992 | 574 | 418 | Up |
| SS_vs_MtS2 | ES | 2312 | 872 | 521 | 351 | Up |
| SS_vs_MtS2 | A5SS | 2028 | 823 | 483 | 340 | Up |
| SS_vs_MtS2 | A3SS | 2869 | 1114 | 650 | 464 | Up |
| SS_vs_MtS2 | MXE | 281 | 107 | 74 | 33 | Up |
| SS_vs_MtS2 | IR | 3364 | 1415 | 785 | 630 | Up |
| SS_vs_MtS3 | ES | 2331 | 983 | 598 | 385 | Up |
| SS_vs_MtS3 | A5SS | 2067 | 920 | 544 | 376 | Up |
| SS_vs_MtS3 | A3SS | 2858 | 1210 | 711 | 499 | Up |
| SS_vs_MtS3 | MXE | 302 | 124 | 84 | 40 | Up |
| SS_vs_MtS3 | IR | 3183 | 1448 | 795 | 653 | Up |
| SS_vs_MtS4 | ES | 2112 | 874 | 535 | 339 | Up |
| SS_vs_MtS4 | A5SS | 1793 | 795 | 483 | 312 | Up |
| SS_vs_MtS4 | A3SS | 2622 | 1128 | 689 | 439 | Up |
| SS_vs_MtS4 | MXE | 264 | 115 | 74 | 41 | Up |
| SS_vs_MtS4 | IR | 2578 | 1129 | 626 | 503 | Up |
| MtS2_vs_MtS1 | ES | 819 | 82 | 38 | 44 | Down |
| MtS2_vs_MtS1 | A5SS | 518 | 54 | 30 | 24 | Up |
| MtS2_vs_MtS1 | A3SS | 678 | 68 | 31 | 37 | Down |
| MtS2_vs_MtS1 | MXE | 102 | 6 | 2 | 4 | Down |
| MtS2_vs_MtS1 | IR | 1233 | 117 | 55 | 62 | Down |
| MtS3_vs_MtS2 | ES | 303 | 10 | 1 | 9 | Down |
| MtS3_vs_MtS2 | A5SS | 203 | 4 | 0 | 4 | Down |
| MtS3_vs_MtS2 | A3SS | 353 | 7 | 4 | 3 | Up |
| MtS3_vs_MtS2 | MXE | 43 | 4 | 3 | 1 | Up |
| MtS3_vs_MtS2 | IR | 622 | 14 | 10 | 4 | Up |
| MtS4_vs_MtS3 | ES | 377 | 5 | 3 | 2 | Up |
| MtS4_vs_MtS3 | A5SS | 225 | 4 | 2 | 2 | Equal |
| MtS4_vs_MtS3 | A3SS | 396 | 3 | 1 | 2 | Down |
| MtS4_vs_MtS3 | MXE | 47 | 0 | 0 | 0 | Equal |
| MtS4_vs_MtS3 | IR | 1022 | 17 | 7 | 10 | Down |

**Note:** AS types include exon skipping (ES), alternative 5′ splice site (A5SS), alternative 3′ splice site (A3SS), mutually exclusive exons (MXE), and intron retention (IR). Dominant DEG direction indicates whether upregulated or downregulated AS-DEGs were more frequent within each AS type.

**Table S37**. Summary of alternative splicing events overlapping with differentially expressed genes across comparisons.

| **Comparison** | **ES** | **A5SS** | **A3SS** | **MXE** | **IR** | **Total** |
| --- | --- | --- | --- | --- | --- | --- |
| SS_vs_MtS1 | 1,241 | 770 | 1,123 | 150 | 1,560 | 4,844 |
| SS_vs_MtS2 | 1,800 | 1,141 | 1,632 | 194 | 2,053 | 6,820 |
| SS_vs_MtS3 | 1,977 | 1,268 | 1,816 | 234 | 2,161 | 7,456 |
| SS_vs_MtS4 | 1,772 | 1,091 | 1,659 | 213 | 1,561 | 6,296 |
| MtS2_vs_MtS1 | 137 | 61 | 81 | 13 | 150 | 442 |
| MtS3_vs_MtS2 | 14 | 4 | 7 | 7 | 19 | 51 |
| MtS4_vs_MtS3 | 17 | 7 | 4 | 0 | 30 | 58 |
