# Supplementary material for: Multi‐Omics Analyses Identify Candidate Genes Associated With Stage‐Specific Quality Formation in Mulching‐Treated Moso Bamboo Shoots
Source: Adv Sci (Weinh). 2026 Oct 30:e78312. Online ahead of print. doi: 10.1002/advs.78312 (PMC13721032; doi:10.1002/advs.78312)
Supplement: Supplementary file 2 [file ADVS-9999-e78312-s002.zip › advs78312-sup-0002-00018-SuppMat/advs78312-sup-00018-S48. Comparison of MtS molecular responses.docx]

*Supplementary Table*

**Multi-omics analyses identify candidate genes associated with stage-specific quality formation in mulching-treated Moso bamboo shoots**

Bo Wang ^1, 3^, Yanbin Wang ^1^, Chunyue Wang ^2^, Zishan Ahmad ^2^, Peixin Teng ^2^, Huijing Ni ^1, 3,^ *, Qiang Wei ^2,^ *****, Muthusamy Ramakrishnan ^2,^ *

^1^ Zhejiang Academy of Forestry, Hangzhou 310023, Zhejiang, China

^2^ State Key Laboratory of Tree Genetics and Breeding, Co-Innovation Center for Sustainable Forestry in Southern China, Bamboo Research Institute, School of Life Sciences, Nanjing Forestry University, Nanjing 210037, Jiangsu, China

^3^ Northwest Zhejiang Bamboo Forest Ecosystem Positioning Observation and Research Station, National Forestry and Grassland Administration, Hangzhou 310023, Zhejiang, China

* Corresponding authors’ emails: [hjni0529@163.com](mailto:hjni0529@163.com) (Huijing Ni); [weiqiang@njfu.edu.cn](mailto:weiqiang@njfu.edu.cn) (Qiang Wei); [ramky@njfu.edu.cn](mailto:ramky@njfu.edu.cn) (Muthusamy Ramakrishnan)

**Table S48.** Comparison of mulching-associated molecular responses in Moso bamboo shoots with established environmental response pathways reported in model plants, including *Arabidopsis* and rice.

| **Potential environmental change associated with mulching** | **Established molecular response in model plants** | **Representative genes and pathways reported** | **Corresponding observations in Moso bamboo shoots (MtS)** | **Interpretation and limitation in the current study (MtS)** | **References** |
| --- | --- | --- | --- | --- | --- |
| Higher temperature | Moderate warming alters growth and transcription through thermomorphogenic signalling, whereas stronger heat activates stress-responsive transcription. In rice, heat stress reprogrammes stress-response, hormone-signalling, metabolic, and protein-processing pathways. | phyB-PIF4-auxin signalling; thermomorphogenesis; HSF-HSP pathways; hormone signalling; carbon metabolism; heat-responsive TFs, including AP2/ERF, NAC, HSF, WRKY, and C2H2 | MtS showed stage-dependent shifts in the bHLH, MYB, NAC, bZIP, C2H2, and SPL families and in carbohydrate- and hormone-related pathways, while *PIF3L6* emerged as a candidate gene. However, our analyses did not identify HSF-HSP signalling or ER protein-processing pathways as major MtS signatures. | MtS shares transcriptional and metabolic features with temperature-responsive networks in Arabidopsis and rice, particularly TF, hormone, and carbon-metabolism responses. However, our analyses did not identify a strong HSF-HSP signature, and we did not measure temperature simultaneously; therefore, we cannot attribute these changes to temperature alone. | (Casal and Balasubramanian 2019; Guo et al. 2016; He et al. 2023; Quint et al. 2016) |
| Lower light availability | Reduced light alters transcription, photosynthetic machinery, and carbon metabolism. In rice, low light changes photosynthesis-related genes and alters light-harvesting and Photosystem I/II components. | Phytochrome-PIF signalling; PIF TFs; photosynthetic and light-harvesting pathways; CAB, SBPase, and Photosystem I/II-related genes | MtS showed stage-dependent *PIF3L6* expression and changes in carbohydrate-related pathways. However, our analyses did not highlight photosynthesis-, light-harvesting-, or phytochrome-associated pathways as major MtS responses. | MtS shares a PIF-related feature with established light-response networks, but our analyses did not highlight the strong photosynthetic and light-harvesting signatures reported under low light in rice. Because we did not measure light above or beneath the mulch, we cannot attribute the MtS patterns directly to reduced light. | (Franklin 2009; Leivar and Monte 2014; Pham et al. 2018; Sekhar et al. 2019) |
| Altered carbon supply and sugar signalling | Changes in carbon availability alter carbohydrate metabolism, while sugars act as metabolic and developmental signals. | Sucrose and starch metabolism; glycolysis; hexokinase- and SnRK-related sugar signalling | MtS accumulated more soluble sugars and showed stage-dependent changes in carbohydrate metabolism. Early and mid-MtS stages showed stronger sugar- and amino-acid-associated quality traits. | These changes indicate carbon reprogramming during MtS development. Temperature, light, and developmental stage may each contribute to this pattern. | (Lastdrager et al. 2014; Rolland et al. 2002; Ruan 2014) |
| Altered secondary metabolism | Environmental and developmental cues reshape phenylpropanoid and flavonoid metabolism and alter MYB- and bHLH-associated networks. | Phenylpropanoid biosynthesis; flavonoid biosynthesis; MYB-bHLH regulatory networks | MtS showed stage-dependent shifts in phenylpropanoid, flavonoid, and tricin-related metabolism. MYB, bHLH, and NAC families also changed across stages. | MtS shares secondary-metabolic patterns with environmentally responsive pathways in model plants, although normal developmental progression may also produce these changes. | (Dong and Lin 2021; Vogt 2010; Xu et al. 2015) |
| Altered hormone-associated growth signalling | Temperature and light cues interact with auxin, gibberellin, ABA, and brassinosteroid signalling to adjust plant growth. | PIF-auxin signalling; GA pathways; ABA signalling; brassinosteroid-PIF interactions | Transcriptomic analyses identified changes in hormone-signalling pathways, while WGCNA and O2PLS prioritised stage-associated candidate genes. | These hormone-related changes may link environmental cues with MtS growth, but the data show association rather than direct environmental regulation. | (Gray et al. 1998; Oh et al. 2012; Pham et al. 2018; Quint et al. 2016) |
| Progressive cell-wall maturation | Developmental networks control cellulose deposition, lignification, and secondary cell-wall formation and respond to environmental cues. | Cellulose synthesis; lignin biosynthesis; NAC-MYB secondary cell-wall networks | Later MtS stages accumulated more cellulose and tannins. Transcriptomic analyses also identified lignin-biosynthesis, cellulose-synthesis, and cell-wall-modification pathways. | These changes mark structural maturation during MtS development. Environmental conditions associated with mulching may modify this trajectory, but the data cannot separate those effects from developmental age. | (Taylor-Teeples et al. 2015; Zhong and Ye 2015) |
| Environmental and developmental regulation of alternative splicing | Environmental and developmental cues alter RNA processing and alternative splicing, changing transcript abundance and isoform use. | Stress-responsive alternative splicing; intron retention; RNA-processing pathways | MtS showed widespread AS-associated DEGs. Intron retention dominated the SS vs MtS1, SS vs MtS2, and SS vs MtS3 comparisons, whereas exon skipping dominated SS vs MtS4. | The AS pattern resembles stress-responsive post-transcriptional changes reported in plants, but the analysis remains discovery-level and does not identify temperature or light as the direct trigger. | (Filichkin et al. 2010; Laloum et al. 2018; Reddy et al. 2013) |

**Note:** The comparisons provide biological context for the MtS and SS multi-omics patterns. WS was not included in the metabolomic and transcriptomic analyses. Therefore, the observed SS-MtS differences may reflect the combined effects of mulching, developmental stage, season, temperature, and light environment, but they do not establish direct temperature- or light-driven effects.

**Supplementary References**

Casal, J.J. and Balasubramanian, S. (2019) Thermomorphogenesis. *Annu. Rev. Plant Biol.* **70**, 321-346. <https://doi.org/10.1146/annurev-arplant-050718-095919>.

Dong, N.-Q. and Lin, H.-X. (2021) Contribution of phenylpropanoid metabolism to plant development and plant-environment interactions. *J. Integr. Plant Biol.* **63**, 180-209. <https://doi.org/10.1111/jipb.13054>.

Filichkin, S.A., Priest, H.D., Givan, S.A., Shen, R., Bryant, D.W., Fox, S.E., Wong, W.K. and Mockler, T.C. (2010) Genome-wide mapping of alternative splicing in *Arabidopsis thaliana*. *Genome Res.* **20**, 45-58. <https://doi.org/10.1101/gr.093302.109>.

Franklin, K.A. (2009) Light and temperature signal crosstalk in plant development. *Curr. Opin. Plant Biol.* **12**, 63-68. <https://doi.org/10.1016/j.pbi.2008.09.007>.

Gray, W.M., Östin, A., Sandberg, G., Romano, C.P. and Estelle, M. (1998) High temperature promotes auxin-mediated hypocotyl elongation in *Arabidopsis*. *Proc. Natl. Acad. Sci. USA* **95**, 7197-7202. <https://doi.org/10.1073/pnas.95.12.7197>.

Guo, M., Liu, J.-H., Ma, X., Luo, D.-X., Gong, Z.-H. and Lu, M.-H. (2016) The plant heat stress transcription factors (HSFs): structure, regulation, and function in response to abiotic stresses. *Front. Plant Sci.* **7**, 114. <https://doi.org/10.3389/fpls.2016.00114>.

He, Y., Guan, H., Li, B., Zhang, S., Xu, Y., Yao, Y., Yang, X., Zha, Z., Guo, Y., Jiao, C. and Cai, H. (2023) Transcriptome analysis reveals the dynamic and rapid transcriptional reprogramming involved in heat stress and identification of heat response genes in rice. *Int. J. Mol. Sci.* **24**, 14802. <https://doi.org/10.3390/ijms241914802>.

Laloum, T., Martín, G. and Duque, P. (2018) Alternative splicing control of abiotic stress responses. *Trends Plant Sci.* **23**, 140-150. <https://doi.org/10.1016/j.tplants.2017.09.019>.

Lastdrager, J., Hanson, J. and Smeekens, S. (2014) Sugar signals and the control of plant growth and development. *J. Exp. Bot.* **65**, 799-807. <https://doi.org/10.1093/jxb/ert474>.

Leivar, P. and Monte, E. (2014) PIFs: systems integrators in plant development. *Plant Cell* **26**, 56-78. <https://doi.org/10.1105/tpc.113.120857>.

Oh, E., Zhu, J.Y. and Wang, Z.Y. (2012) Interaction between *BZR1* and *PIF4* integrates brassinosteroid and environmental responses. *Nat. Cell Biol.* **14**, 802-809. <https://doi.org/10.1038/ncb2545>.

Pham, V.N., Kathare, P.K. and Huq, E. (2018) Phytochromes and phytochrome interacting factors. *Plant Physiol.* **176**, 1025-1038. <https://doi.org/10.1104/pp.17.01384>.

Quint, M., Delker, C., Franklin, K.A., Wigge, P.A., Halliday, K.J. and van Zanten, M. (2016) Molecular and genetic control of plant thermomorphogenesis. *Nat. Plants* **2**, 15190. <https://doi.org/10.1038/nplants.2015.190>.

Reddy, A.S.N., Marquez, Y., Kalyna, M. and Barta, A. (2013) Complexity of the alternative splicing landscape in plants. *Plant Cell* **25**, 3657-3683. <https://doi.org/10.1105/tpc.113.117523>.

Rolland, F., Moore, B. and Sheen, J. (2002) Sugar sensing and signaling in plants. *Plant Cell* **14**, S185-S205. <https://doi.org/10.1105/tpc.010455>.

Ruan, Y.-L. (2014) Sucrose metabolism: gateway to diverse carbon use and sugar signaling. *Annu. Rev. Plant Biol.* **65**, 33-67. <https://doi.org/10.1146/annurev-arplant-050213-040251>.

Sekhar, S., Panda, D., Kumar, J., Mohanty, N., Biswal, M., Baig, M.J., Kumar, A., Umakanta, N., Samantaray, S., Pradhan, S.K., Shaw, B.P., Swain, P. and Behera, L. (2019) Comparative transcriptome profiling of low light tolerant and sensitive rice varieties induced by low light stress at active tillering stage. *Sci. Rep.* **9**, 5753. <https://doi.org/10.1038/s41598-019-42170-5>.

Taylor-Teeples, M., Lin, L., de Lucas, M., Turco, G., Toal, T.W., Gaudinier, A., Young, N.F., Trabucco, G.M., Veling, M.T., Lamothe, R., Handakumbura, P.P., Xiong, G., Wang, C., Corwin, J., Tsoukalas, A., Zhang, L., Ware, D., Pauly, M., Kliebenstein, D.J., Dehesh, K., Tagkopoulos, I., Breton, G., Pruneda-Paz, J.L., Ahnert, S.E., Kay, S.A., Hazen, S.P. and Brady, S.M. (2015) An *Arabidopsis* gene regulatory network for secondary cell wall synthesis. *Nature* **517**, 571-575. <https://doi.org/10.1038/nature14099>.

Vogt, T. (2010) Phenylpropanoid biosynthesis. *Mol. Plant* **3**, 2-20. <https://doi.org/10.1093/mp/ssp106>.

Xu, W., Dubos, C. and Lepiniec, L. (2015) Transcriptional control of flavonoid biosynthesis by MYB-bHLH-WDR complexes. *Trends Plant Sci.* **20**, 176-185. <https://doi.org/10.1016/j.tplants.2014.12.001>.

Zhong, R. and Ye, Z.-H. (2015) Secondary cell walls: biosynthesis, patterned deposition and transcriptional regulation. *Plant Cell Physiol.* **56**, 195–214. <https://doi.org/10.1093/pcp/pcu140>.
