# Supplementary material for: Multi‐Omics Analyses Identify Candidate Genes Associated With Stage‐Specific Quality Formation in Mulching‐Treated Moso Bamboo Shoots
Source: Adv Sci (Weinh). 2026 Oct 30:e78312. Online ahead of print. doi: 10.1002/advs.78312 (PMC13721032; doi:10.1002/advs.78312)
Supplement: Supplementary file 2 [file ADVS-9999-e78312-s002.zip › advs78312-sup-0002-00018-SuppMat/advs78312-sup-0002-S1. List of primers used for qRT-PCR analysis.docx]

*Supplementary Table*

**Multi-omics analyses identify candidate genes associated with stage-specific quality formation in mulching-treated Moso bamboo shoots**

Bo Wang ^1, 3^, Yanbin Wang ^1^, Chunyue Wang ^2^, Zishan Ahmad ^2^, Peixin Teng ^2^, Huijing Ni ^1, 3,^ *, Qiang Wei ^2,^ *****, Muthusamy Ramakrishnan ^2,^ *

^1^ Zhejiang Academy of Forestry, Hangzhou 310023, Zhejiang, China

^2^ State Key Laboratory of Tree Genetics and Breeding, Co-Innovation Center for Sustainable Forestry in Southern China, Bamboo Research Institute, School of Life Sciences, Nanjing Forestry University, Nanjing 210037, Jiangsu, China

^3^ Northwest Zhejiang Bamboo Forest Ecosystem Positioning Observation and Research Station, National Forestry and Grassland Administration, Hangzhou 310023, Zhejiang, China

* Corresponding authors’ emails: [hjni0529@163.com](mailto:hjni0529@163.com) (Huijing Ni); [weiqiang@njfu.edu.cn](mailto:weiqiang@njfu.edu.cn) (Qiang Wei); [ramky@njfu.edu.cn](mailto:ramky@njfu.edu.cn) (Muthusamy Ramakrishnan)

**Table S1**. List of primers used for qRT-PCR analysis.

| **Gene name** | **Primer name** | **Primer sequence (5′-3′)** | **Primer length (bp)** |
| --- | --- | --- | --- |
| *PH02Gene37115* (*ATH1L*) | 37115-F | GCCTGCTCGGTGGACTTACA | 20 |
|  | 37115-R | TGGATTGAGGTAGCGTTTGGTC | 22 |
| *PH02Gene08257* (*PIF3L6*) | 08257-F | AACAATGTCATCTGCGAGCCA | 21 |
|  | 08257-R | CATCAGAGGAAGACGGTGGCT | 21 |
| *PH02Gene08434* (*NID1*) | 08434-F | TCAAGACGGAAGAGGAGGTTAAG | 23 |
|  | 08434-R | CCTTCTGCTGCCCAAAATGTA | 21 |
| *PH02Gene30756* (*SPL18*) | 30756-F | GGAAGCCACAGCCAGATAACA | 21 |
|  | 30756-R | CCTCAGATTTGATGATCCCAGAC | 23 |
| *PH02Gene32400* (*TOM6*) | 32400-F | TCTGCCGATGTCGTCGTCTAA | 21 |
|  | 32400-R | GCCACCAATGCCTCCGTT | 18 |
